# Inferring the COVID-19 infection fatality rate in the community-dwelling population: a simple Bayesian evidence synthesis of seroprevalence study data and imprecise mortality data

**DOI:** 10.1017/S0950268821002405

**Published:** 2021-11-08

**Authors:** Harlan Campbell, Paul Gustafson

**Affiliations:** Department of Statistics, University of British Columbia, Vancouver, British Columbia, Canada

**Keywords:** COVID-19, evidence synthesis, Bayesian inference, infection fatality rate

## Abstract

Estimating the coronavirus disease-2019 (COVID-19) infection fatality rate (IFR) has proven to be particularly challenging –and rather controversial– due to the fact that both the data on deaths and the data on the number of individuals infected are subject to many different biases. We consider a Bayesian evidence synthesis approach which, while simple enough for researchers to understand and use, accounts for many important sources of uncertainty inherent in both the seroprevalence and mortality data. With the understanding that the results of one's evidence synthesis analysis may be largely driven by which studies are included and which are excluded, we conduct two separate parallel analyses based on two lists of eligible studies obtained from two different research teams. The results from both analyses are rather similar. With the first analysis, we estimate the COVID-19 IFR to be 0.31% [95% credible interval (CrI) of (0.16%, 0.53%)] for a typical community-dwelling population where 9% of the population is aged over 65 years and where the gross-domestic-product at purchasing-power-parity (GDP at PPP) per capita is $17.8k (the approximate worldwide average). With the second analysis, we obtain 0.32% [95% CrI of (0.19%, 0.47%)]. Our results suggest that, as one might expect, lower IFRs are associated with younger populations (and may also be associated with wealthier populations). For a typical community-dwelling population with the age and wealth of the United States we obtain IFR estimates of 0.43% and 0.41%; and with the age and wealth of the European Union, we obtain IFR estimates of 0.67% and 0.51%.

Above all, what's needed is humility in the face of an intricately evolving body of evidence. The pandemic could well drift or shift into something that defies our best efforts to model and characterise it.

Siddhartha Mukherjee, *The New Yorker*

22 February 2021

## Introduction

The infection fatality ratio (IFR), defined as the proportion of individuals infected who will go on to die as a result of their infection, is a crucial statistic for understanding severe acute respiratory syndrome coronavirus 2 (SARS-CoV-2) and the ongoing coronavirus disease-2019 (COVID-19) pandemic. Estimating the COVID-19 IFR has proven to be particularly challenging –and rather controversial– due to the fact that both the data on deaths and the data on the number of individuals infected are subject to many different biases.

SARS-CoV-2 seroprevalence studies can help provide a better understanding of the true number of infections in a given population and for this reason, several researchers have sought to leverage seroprevalence study data to infer the COVID-19 IFR [1]. In particular, Ioannidis [2], Levin *et al*. [3], Brazeau *et al*. [4] and O'Driscoll *et al*. [5] have all undertaken analyses, of varying degrees of complexity, in which they combine data from multiple seroprevalence studies with available mortality statistics to derive IFR estimates.

The analyses of both Brazeau *et al*. [4] and O'Driscoll *et al*. [5] are done using rather complex Bayesian models which rely on numerous detailed assumptions. For instance, Brazeau *et al*. [4] use a Bayesian ‘statistical age-based model that incorporates delays from onset of infection to seroconversion and onset of infection to death, differences in IFR and infection rates by age and the uncertainty in the serosample collection time and the sensitivity and specificity of serological tests.’ O'Driscoll *et al*. [5] employ a Bayesian ensemble model which assumes ‘a gamma-distributed delay between onset [of infection] and death’ and assumes different risks of infection for ‘individuals aged 65 years and older, relative to those under 65.’ While these analyses go to great lengths to account for the various sources of uncertainty in the data, the complexity of the models will no doubt make it challenging for other researchers to fit these models to different data in a constantly evolving pandemic.

In contrast, the analyses of Ioannidis [2] and Levin *et al*. [3] are decidedly more simple. For each seroprevalence study under consideration, Ioannidis [2] counts the cumulative number of deaths (from the beginning of the pandemic) until 7 days after the study mid-point (or until the date the study authors suggest) and divides this number of deaths by the estimated number of (previous or current) infections to obtain a study-specific IFR estimate. A ‘location specific’ IFR estimate is then obtained by taking a weighted (by the study's sample size) average of the study-specific IFR estimates for a given location (i.e. for a given country or state). Ioannidis [2] then calculates the median of all the location-specific IFR estimates. No uncertainty interval for this estimate is provided. As such, it is impossible to determine what level of confidence one should place in Ioannidis [2]'s estimates.

The analysis of Levin *et al*. [3] is based on a standard frequentist random-effects meta-analysis model. For each age-group and seroprevalence study under consideration, Levin *et al*. [3] calculate a 95% confidence interval (CI) for a study-specific IFR by counting the cumulative number of deaths up until 4 weeks after the study mid-point and dividing this number of deaths by the estimated upper and lower bounds of the number of infected individuals. The meta-analysis model then combines each of these study-specific IFRs. While this analysis provides standard confidence intervals and is relatively straightforward, it does not take into account certain important sources of uncertainty (to be discussed in Section ‘methods’).

The analysis method we propose is simple enough for researchers to easily understand and use, while accounting for important sources of uncertainty inherent in both the seroprevalence data and the mortality data. Similar Bayesian models have been used previously for evidence synthesis of seroprevalence data for other infectious diseases (e.g. Brody-Moore [6]). We will apply the method in analysis with the objective of estimating the average COVID-19 IFR in a community-dwelling population with a certain approximate age composition and wealth.

A major part in any evidence synthesis is determining which studies to consider within the analysis. Determining appropriate inclusion and exclusion criteria for seroprevalence studies is a rather complicated and delicate issue when it comes to estimating the COVID-19 IFR [7, 8]. Reviewing and evaluating the merits of the hundreds of available seroprevalence studies also involves a tremendous amount of review work and time. Fortunately, both Chen *et al*. [9] and Arora *et al*. [10] have done comprehensive and thorough reviews to ascertain study quality (i.e. risk of bias). We will work from these two lists to conduct two separate parallel analyses. This approach –conducting two analyses based on two distinct and independent literature reviews– will allow us to better understand the impact of different inclusion and exclusion criteria [11]. We will review the data and how it was obtained following a review of the methods.

## Methods

### Bayesian Model for evidence synthesis

Suppose we have data from *K* different seroprevalence studies. Then, for *k* = 1, …, *K*, let:

- *T*_*k*_ be the total number of individuals tested in the *k*-th study;
- *CC*_*k*_ be the total number of confirmed cases (of past or current infection) resulting from those tested in the *k*-th study;
- *P*_*k*_ be the number of individuals at risk of infection in the population of interest for the *k*-th study; and
- *D*_*k*_ be the total number of observed deaths (cumulative since pandemic onset) in the population of interest that are attributed to infection.

We do not observe the following latent (i.e. unknown) variables; for *k* = 1, …, *K*, let:

- *C*_*k*_ be the total number of infected people (cases) in the *k*-th population;
- IR_*k*_ be the true infection rate (proportion of the *k*-th population which has been infected), which is the expected value of *C*_*k*_/*P*_*k*_; and
- IFR_*k*_ be the true underlying infection fatality rate (IFR), which is the expected value of *D*_*k*_/*C*_*k*_ (given *C*_*k*_).

We will make a series of simple binomial assumptions such that, for *k* = 1, …, *K*:1
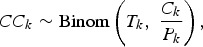
2
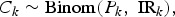
3
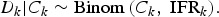


We wish to emphasise the importance of the third ‘*D*|*C*’ binomial distribution above. Failing to account for the conditional distribution of the deaths given the cases may lead to inappropriately precise estimates of the IFR. For example, Streeck *et al*. [12] (in their original preprint (*medRxiv*, May 8, 2020)) calculate an uncertainty interval for the IFR by dividing the number of deaths (*D* = 7) by the upper and lower bounds of the 95% confidence interval (CI) for the number of infections (95% CI for *C* = [1551, 2389]). Doing so, they obtain a relatively narrow 95% CI for the IFR: [0.29%, 0.45%] (=[7/1,551, 7/2389]). In the published version of their article (*Nature Communications*, November 17, 2020), an alternative interval “accounting for uncertainty in the number of recorded deaths” is provided. This alternative interval, which essentially takes into account the *D*|*C* binomial distribution, is substantially wider: [0.17%; 0.77%]. In a very similar way, Levin *et al*. [3] also fail to take into account the *D*|*C* binomial distribution when estimating study-specific IFRs resulting in spuriously precise study-specific IFR estimates.

Having established simple binomial distributions for the study-specific IRs and IFRs, we define a simple random-effects model such that, for *k* = 1, …, *K*:4
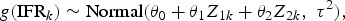
and5
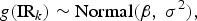
where *θ*_0_ represents the mean g(IFR), *τ*^2^ represents between-group IFR heterogeneity, *β* represents the mean g(infection rate), *σ*^2^ describes the variability in infection rates across the *K* groups, *Z*_1*k*_ and *Z*_2*k*_ are covariates of interest that may be related to the IFR by means of the *θ*_1_ and *θ*_2_ parameters and g() is a given link function. In our analysis, we define g() as the complimentary log-log link function (cloglog), though there are other sensible choices including the logit and probit functions. As for the two covariates, *Z*_1*k*_ and *Z*_2*k*_, we will define these as the centred and scaled logarithm of the proportion of the population aged over 65 years (65 yo_*k*_) and of the GDP [at purchasing power parity (PPP)] per capita (GDP_*k*_), respectively.

The model is considered within a Bayesian framework requiring the specification of priors for the unknown parameters. Our strategy for priors is to assume weakly informative priors. Beta, Normal and half-Normal priors (following the recommendations of Gelman *et al*. [13] and Kümmerer *et al*. [14]) are set accordingly: *g*^−1^(*θ_0_*) ~ Beta(0.3, 3); *g*^−1^(*β*) ~ Beta(1, 30); 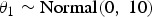; 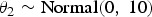; 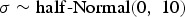; and 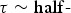 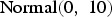. Note that the performance of any Bayesian estimator will depend on the choice of priors and that this choice can substantially influence the posterior when few data are available [15, 16]. In the Supplementary Material, we show results obtained with an alternative set of priors as a sensitivity analysis.

### Uncertainty in infection rates

While some seroprevalence studies report the exact number of individuals tested and the exact number of confirmed cases amongst those tested, to obtain estimates for the infection rate there are typically numerous adjustments made (e.g. adjusting for imperfect diagnostic test accuracy, adjusting for clustering of individuals within a household). For this reason, the sample size of a given study might not be a reliable indicator of its precision and weighting a study's contribution in an evidence synthesis based solely on its sample size (as in e.g. Ioannidis [2]) may not be appropriate.

Rather than work with the raw testing numbers published in the seroprevalence studies, we calculate effective data values for *T*_*k*_ and *CC*_*k*_ based on a binomial distribution that corresponds to the reported 95% CI for the IR. By ‘inverting uncertainty intervals’ in this way, we are able to properly use the adjusted numbers provided. (This is a similar approach to the strategy employed by Kümmerer *et al*. [14].) Tables 1 and 2 list the 95% uncertainty intervals obtained from each of the seroprevalence studies in our two parallel analyses and Tables 3 and 4 list the corresponding values for *T*_*k*_ and *CC*_*k*_.

**Table 1.** Seroprevalence studies selected for the analysis based on the list compiled by Chen *et al*. [9] (listed in alphabetical order of authors), with the geographic location of sampling, sampling dates and 95% uncertainty interval for the infection rate (IR interval). Also noted, under ‘In both analyses’, is whether or not each study is included in the Serotracker-based analysis (i.e. is also in Table 2). Studies with yes* are alternate versions of studies that are included in the Serotracker-based analysis. Note that sampling for all studies took place during mid-2020, before the widespread availability of COVID-19 vaccinations.

Authors	Location	Sampling (mm/dd)	IR interval (%)	In both analyses
Barchuk *et al*. [17]	Saint Petersburg, Russia	05/27–06/26	(5.60, 12.90)	yes
Biggs *et al*. [18]	DeKalb and Fulton, GA, USA (A)	04/28–05/03	(1.40, 4.50)	yes*
Bruckner *et al*. [19]	Orange County, CA, USA	07/10–08/16	(8.10, 15.50)	yes
Carrat *et al*. [20]	Ile-de-France, France	05/04–06/14	(8.90, 11.30)	
Mahajan *et al*. [21]	Connecticut, USA	06/10–07/29	(1.70, 6.30)	
Murhekar *et al*. (A) [22]	India	05/11–06/04	(0.34, 1.13)	yes
Office of National Stat [23]	England, UK (A)	04/26–09/08	(5.40, 7.10)	
Petersen *et al*. [24]	Faroe Islands, Denmark	04/27–05/01	(0.10, 1.20)	yes
Pollan *et al*. [25]	Spain	04/27–05/11	(3.30, 6.60)	yes
Samore *et al*. [26]	Four counties in UT, USA	05/04–06/30	(0.10, 1.60)	
Santos-Hovener *et al*. [27]	Kupferzell, Germany	05/20–06/09	(10.40, 14.00)	yes
Sharma *et al*. [28]	Delhi, India	08/01–08/07	(27.65, 29.14)	yes
Snoeck *et al*. [29]	Luxembourg	04/15–05/05	(1.23, 2.77)	
Sood *et al*. [30]	Los Angeles County, CA, USA	04/10–04/14	(2.52, 7.07)	
Statistics Jersey (A) [31]	Jersey, UK (A)	04/29–05/05	(1.80, 4.40)	yes*
Streeck *et al*. [12]	Gangelt, Germany	03/31–04/06	(12.31, 24.40)	yes
Stringhini *et al*. (A) [32]	Geneva, Switzerland (A)	04/06–05/09	(8.15, 13.95)	yes*
Vos *et al*. [33]	Netherlands	03/31–05/11	(2.10, 3.70)	yes
Ward *et al*. [34]	England, UK (B)	06/20–07/13	(5.78, 6.14)	yes

**Table 2.** Seroprevalence studies selected for the analysis based on the list compiled by Serotracker (listed in alphabetical order of authors), with the geographic location of sampling, sampling dates and 95% uncertainty interval for the infection rate (IR interval). Also noted, under ‘In both analyses’, is whether or not each study is included in the Serotracker-based analysis (i.e. is also in Table 2). Studies with yes* are alternate versions of studies that are included in the Serotracker-based analysis. Note that sampling for all studies took place during mid-2020, before the widespread availability of COVID-19 vaccinations.

Authors	Location	Sampling (mm/dd)	IR interval (%)	In both analyses
Álvarez-Antonio *et al*. [35]	Iquitos, Peru	07/13–07/18	(67.00, 73.00)	
Bajema *et al*. [36]	DeKalb and Fulton, GA, USA (B)	04/28–05/03	(1.49, 6.67)	yes*
Barchuk *et al*. [17]	Saint Petersburg, Russia	05/27–06/26	(5.60, 12.90)	yes
Bruckner *et al*. [19]	Orange County, CA, USA	07/10–08/16	(8.10, 15.50)	yes
Chan *et al*. [37]	Rhode Island, USA	05/05–05/22	(1.00, 6.20)	
Gov. of Andorra [38]	Andorra	05/04–05/28	(10.50, 11.50)	
Kar *et al*. [39]	Puducherry District, India	08/11–08/16	(3.50, 6.40)	
Khalagi *et al*. [40]	Iran	08/03–10/31	(13.30, 15.20)	
Malani *et al*. [41]	Tamil Nadu, India	10/19–11/30	(30.40, 32.80)	
Melotti *et al*. [42]	Gardena Valley, Italy	05/26–06/08	(25.20, 28.60)	
MoHoI [43]	Israel	06/28–09/17	(5.30, 5.60)	
Murhekar *et al*. (A) [22]	India	05/11–06/04	(0.34, 1.13)	yes
Nawa *et al*. [44]	Utsunomiya City, Japan	06/14–07/05	(0.08, 2.28)	
Pagani *et al*. [45]	Castiglione d'Adda, Italy	05/18–06/07	(17.20, 29.10)	
Petersen *et al*. [24]	Faroe Islands, Denmark	04/27–05/01	(0.10, 1.20)	yes
Pollan *et al*. [25]	Spain	04/27–05/11	(3.30, 6.60)	yes
Radon *et al*. [46]	Munich, Germany	11/02–01/31	(2.90, 4.30)	
Reyes-Vega *et al*. [47]	Lima, Peru	06/28–07/09	(22.50, 28.20)	
Richard *et al*. [48]	Geneva, Switzerland (B)	04/06–06/30	(6.80, 8.90)	yes*
Santos-Hovener *et al*. [27]	Kupferzell, Germany	05/20–06/09	(10.40, 14.00)	yes
Selvaraju *et al*. [49]	Chennai, Tamil Nadu, India	07/01–07/31	(14.80, 22.60)	
Sharma *et al*. [28]	Delhi, India	08/01–08/07	(27.65, 29.14)	yes
Statistics Jersey (B) [50]	Jersey, UK (B)	06/21–06/27	(2.80, 5.20)	yes*
Streeck *et al*. [12]	Gangelt, Germany	03/31–04/06	(12.31, 24.40)	yes
Vos *et al*. [33]	Netherlands	03/31–05/11	(2.10, 3.70)	yes
Ward *et al*. [34]	England, UK (B)	06/20–07/13	(5.78, 6.14)	yes
Warszawski *et al*. [51]	Metropolitan France	05/02–06/02	(3.90, 5.00)	
Yoshiyama *et al*. [52]	Tokyo, Japan	06/01–06/07	(0.01, 0.37)

**Table 3.** The Chen *et al*. based dataset required for the Bayesian evidence synthesis model

		*D*_*k*_	*D*_*k*_			65*yo*_*k*_	*GDP*_*k*_
Location	*P*_*k*_	lower	upper	*T*_*k*_	*CC*_*k*_	(%)	($)
Saint Petersburg, Russia	5 351 935	1596	5128	233	21	18	30 144
DeKalb and Fulton, GA, USA (A)	1 823 234	122	136	419	11	12	58 933
Orange County, CA, USA	3 010 232	512	839	285	33	15	79 287
Ile-de-France, France	12 213 447	6816	7427	2414	243	14	82 574
Connecticut, USA	2 837 877	1136	1179	251	9	22	80 729
India	1 366 417 750	4172	45 766	1632	11	6	4735
England, UK (A)	56 550 000	25 975	30 709	3100	193	18	43 310
Faroe Islands, Denmark	52 154	0	0	592	3	17	60 421
Spain	47 351 567	17 710	17 767	643	31	20	42 362
Four counties in UT, USA	2 194 298	40	97	393	2	10	60 050
Kupferzell, Germany	6247	1	3	1263	153	16	63 885
Delhi, India	19 800 000	4188	26 901	13 966	3965	4	12 817
Luxembourg	632 275	47	58	1214	23	14	122 166
Los Angeles County, CA, USA	10 039 107	529	606	316	14	14	79 287
Jersey, UK (A)	107 800	17	18	648	19	17	48 365
Gangelt, Germany	12 597	8	8	153	27	18	53 751
Geneva, Switzerland (A)	504 128	122	155	442	48	16	68 964
Netherlands	17 344 874	2621	4186	1652	47	20	59 685
England, UK (B)	56 550 000	29 526	30 038	10 635	634	18	43 310

**Table 4.** The Serotracker-based dataset required for the Bayesian evidence synthesis model

		*D*_*k*_	*D*_*k*_			65*yo*_*k*_	*GDP*_*k*_
Location	*P*_*k*_	lower	upper	*T*_*k*_	*CC*_*k*_	(%)	($)
Iquitos, Peru	467 000	1581	1942	895	626	4	5896
DeKalb and Fulton, GA, USA (B)	1 823 234	122	136	196	7	12	58 933
Saint Petersburg, Russia	5 351 935	1596	5128	233	21	18	30 144
Orange County, CA, USA	3 010 232	512	839	285	33	15	79 287
Rhode Island, USA	1 059 361	147	189	163	5	18	58 416
Andorra	77 543	35	35	11 236	1235	17	49 900
Puducherry District, India	1 250 000	145	393	840	41	6	9152
Iran	82 913 906	19 804	98 071	5197	740	6	12 937
Tamil Nadu, India	83 697 770	11 183	29 773	5765	1821	7	7191
Gardena Valley, Italy	10 700	15	15	2612	702	20	71 853
Israel	9 216 900	187	995	10 578	577	11	40 747
India	1 366 417 750	4172	45 766	1632	11	6	4735
Utsunomiya City, Japan	517 527	0	0	228	2	26	42 931
Castiglione d'Adda, Italy	4605	39	40	190	43	26	59 291
Faroe Islands, Denmark	52 154	0	0	592	3	17	60 421
Spain	47 351 567	17 710	17 767	643	31	20	42 362
Munich, Germany	1 563 090	219	780	2692	96	17	65 345
Lima, Peru	10 804 609	21 109	28 846	893	226	9	19 313
Geneva, Switzerland (B)	504 128	122	156	2509	196	16	68 964
Kupferzell, Germany	6247	1	3	1263	153	16	63 885
Chennai, Tamil Nadu, India	10 900 000	1295	5960	380	70	7	7191
Delhi, India	19 800 000	4188	26 901	13 966	3965	4	12 817
Jersey, UK (B)	107 800	20	20	998	39	17	48 365
Gangelt, Germany	12 597	8	8	153	27	18	53 751
Netherlands	17 344 874	2621	4186	1652	47	20	59 685
England, UK (B)	56 550 000	29 526	30 038	10 635	634	18	43 310
Metropolitan France	64 897 954	17 396	19 038	5377	238	21	49 551
Tokyo, Japan	13 960 236	270	275	1314	2	20	45 796

It must be noted that, as Ioannidis [2] cautions, it is possible that under our ‘inverting uncertainty intervals’ approach, poorly conducted seroprevalence studies which fail to make proper adjustments (and thereby have spuriously narrower uncertainty intervals) receive more weight in our analysis, while high-quality studies, which make proper adjustments, are unfairly penalised. Ioannidis [2] notes that the strategy of ‘weighting the study-specific IFRs by the sample size of each study’ avoids giving more weight to studies ‘with seemingly narrower confidence intervals because of poor or no adjustments, while still giving more weight to larger studies.’ Since we are restricting our analysis to only those supposedly high-quality seroprevalence studies, we hope to largely avoid this issue. Weighting studies based on their true precision is obviously the goal in any evidence synthesis and we recognise that this is particularly difficult when so many studies may misrepresent the precision of their estimates [53, 54].

### Uncertainty in mortality

Matching prevalence estimates with a relevant number of fatalities is a difficult task. Prevalence estimates obtained from a seroprevalence study do not typically correspond to a specific date. Instead, these estimates will correspond to a window of time during which testing occurred. This period may be only a few days for some studies (e.g. 4 days for Petersen *et al*. [24]), but can also be several weeks or months for others (e.g. 135 days for Ward *et al*. [34]). Tables 1 and 2 list the sampling window start and end dates for each of the studies in our two parallel analyses.

Evidently, a longer sampling window will lead to greater uncertainty when it comes to establishing the relevant number of deaths. It can be difficult to account for this uncertainty and analyses will often simply select a specific date at which to count deaths based on some simple rule of thumb. For example, Ioannidis [2] considers the number of deaths at 7 days after the mid-point of the sampling window (or as the relevant number of deaths discussed by the seroprevalence study's authors). As another example, Meyerowitz-Katz and Merone [55] take the number of deaths as recorded at 10 days after the end of the sampling window. While these two particular analytical choices are not all that different, each may lead to a substantially different number of deaths for a given study if the study was conducted during a period of time during which the number of deaths was rapidly accelerating. Levin *et al*. [3], who consider the number of deaths up until 4 weeks after the sampling window mid-point, acknowledge this limitation noting that: ‘matching prevalence estimates with subsequent fatalities is not feasible if a seroprevalence study was conducted in the midst of an accelerating outbreak.’

In order to account for the uncertainty in selecting the relevant number of deaths for a given seroprevalence study, we propose considering the number of deaths as interval-censored data. Tables 3 and 4 list numbers for an interval corresponding to the number of deaths recorded 14 days after the start of the sampling window and 14 days after the end of the sampling window for each seroprevalence study. While we might not know exactly what number of deaths is most appropriate, we can be fairly confident that the appropriate number lies somewhere within this interval. (Note that some intervals in Tables 3 and 4 have also been widened to account for other sources of uncertainty in the number of deaths; see details in the Supplementary Material.) The 14-day offset allows for the known delay between the onset of infection and death, taking into consideration the delay between the onset of infection and the development of detectable antibodies [56, 57].

## The data

### Seroprevalence data

As the COVID-19 pandemic has progressed, a rapidly increasing number of SARS-CoV-2 seroprevalence studies have been conducted worldwide [10]. However, many of these studies have produced biased estimates or are otherwise unreliable due to a variety of different issues with study design and/or with data collection and/or with inappropriate statistical analysis [53]. We seek to restrict our analysis to high-quality studies which used probability-based sampling methods. Such studies are less likely to suffer from substantial biases [58]. Based on the reviews of Chen *et al*. [9] and of Arora *et al*. [10], we compiled two separate sets of studies for analysis (these are listed in Tables 1 and 2, respectively). With the understanding that the results of an evidence synthesis may be largely driven by which studies are included/excluded, we will use these two separate sets to conduct two separate analyses. Note that the data collected for both analyses are relevant to the time before the widespread availability of COVID-19 vaccinations.

**Fig. 1.**
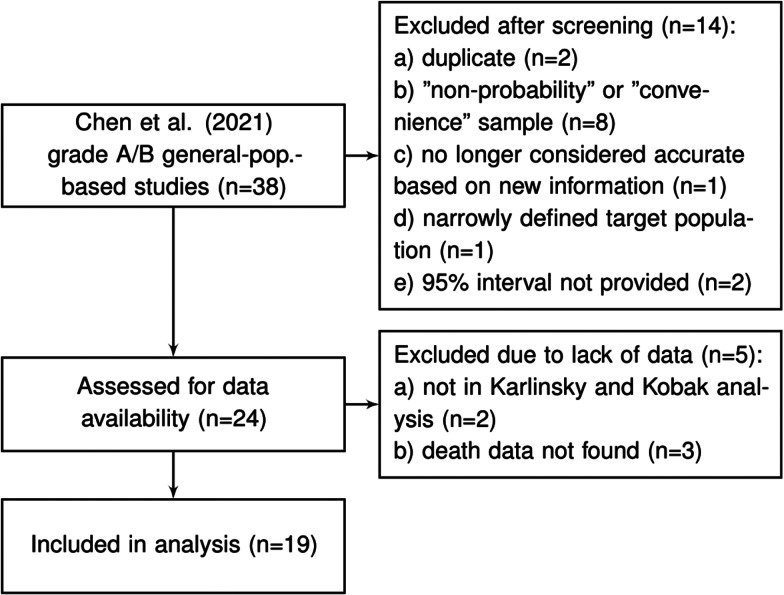
Flowchart of seroprevalence studies considered for Chen *et al*. based analysis.

Chen *et al*. [9] reviewed the literature for articles published between 1 December 2019 and 22 December 2020 and identified more than 400 unique seroprevalence studies. For each of these, study quality was established using a scoring system developed on the basis of a seroepidemiological protocol from the Consortium for the Standardization of Influenza Seroepidemiology [59]. In total, Chen *et al*. [9] identified 38 articles which considered a sample based on a ‘general population’ and which obtained a study quality grade of A or B (see the full list in Supplementary Table S8 of Chen *et al*. [9]). We consider these 38 articles as a starting point for inclusion for our analysis. After excluding those studies which are duplicates (*n* = 2), those that used a ‘convenience’ or ‘non-probability’ based sampling method (according to the classification of Arora *et al*. [10]) (*n* = 8), a study no longer considered accurate based on new information about the accuracy of the antibody test used (*n* = 1), a study that has a very narrowly defined target population (*n* = 1), studies for which relevant death data could not be found (*n* = 5) and studies which did not provide a 95% uncertainty interval (*n* = 2), we were left with a set of *K* = 19 studies for analysis; see Figure 1 and details in the Supplementary Material.

**Fig. 2.**
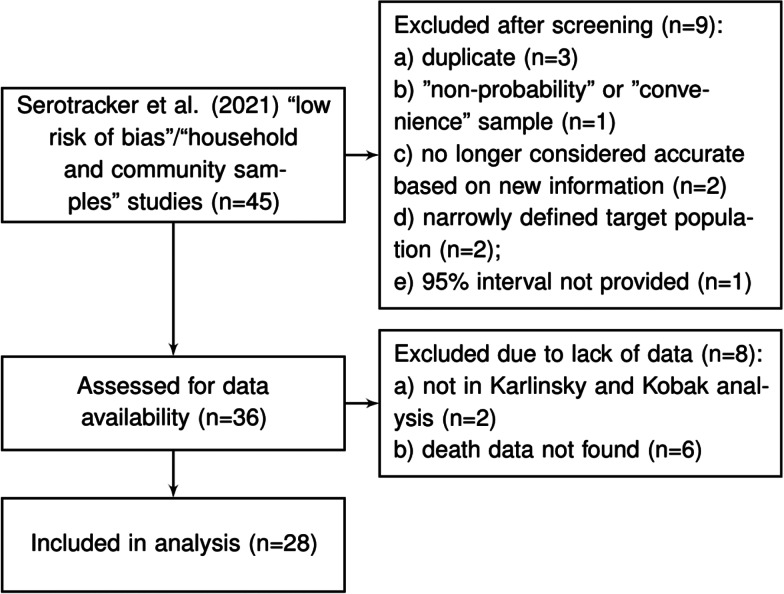
Flowchart of seroprevalence studies considered for Serotracker-based analysis.

Arora *et al*. [10] conducted the Serotracker ‘living systematic review’ of COVID-19 seroprevalence studies whereby the results of the review are continuously updated on serotracker.com/data. For each study reviewed, the risk of bias was evaluated based on an assessment using the Joanna Briggs Institute Critical Appraisal Guidelines for Prevalence studies [53, 60]. For analysis, we consider the 45 studies listed on serotracker.com/data (as of 5 June 2021), that are categorised as having a ‘low risk of bias’ and are categorised as targeting ‘household and community samples.’ After excluding those studies which are duplicates (*n* = 3), one study that used a ‘convenience’ or ‘non-probability’ based sampling method (according to the classification of Arora *et al*. [10]) (*n* = 1), those studies no longer considered accurate based on new information about the accuracy of the antibody test used (*n* = 2), those that have very narrowly defined target populations (*n* = 2), those for which relevant death data could not be found (*n* = 8) and those which did not provide a 95% uncertainty interval for the estimated prevalence (*n* = 1), we are left with a set of *K* = 28 studies for analysis; see Figure 2 and details in the Supplementary Material.

For each of the seroprevalence studies included in each of the two analysis sets, we recorded the 95% uncertainty interval for the infection rate as reported in the study article. If an article reported on multiple phases of a study (e.g. a longitudinal series of different surveys), or reported different results for different areas instead of an overall estimate (e.g. a series of different estimates for different regions), we selected only the first set of estimates. Furthermore, if a study reported more than one 95% uncertainty interval (e.g. different intervals corresponding to different adjustments and assumptions), we selected the lowest value amongst the different lower bounds and the highest value amongst the different upper bounds. These numbers are recorded in Tables 1 and 2 under *IR interval*. Based on these numbers, we calculated effective data values for the number of tests (*T*_*k*_) and the number of confirmed cases (*CC*_*k*_) which are listed in Tables 3 and 4 alongside population numbers (*P*_*k*_) and numbers corresponding to the proportion of the population over 65 years old (65 yo_*k*_) and the GDP (PPP) per capita (GDP_*k*_); see Supplemental Material for details and data sources.

### Mortality Data

Mortality data were obtained from various sources (e.g. academic, government, health authority); see details in Supplementary Material. If a seroprevalence study referenced a specific source for mortality data, we used the referenced source for our numbers whenever possible. If no source was referenced or suggested, we considered publicly available data sources.

For many populations, there were concerns that cause of death information may be very inaccurate and lead to biased COVID-19 mortality statistics. To overcome this issue, many have suggested looking to ‘excess deaths’ by comparing aggregate data for all-cause deaths from the time during the pandemic to the years prior [61]. For populations with a large discrepancy between the ‘official’ number of deaths attributed to COVID-19 and the number of excess deaths –as suggested, when possible, by a large undercount ratio (UCR) derived by Karlinsky and Kobak [62]– we used the ‘official’ number of deaths attributed to COVID-19 for the lower bound of the *D*_*k*_ interval and used numbers based on excess deaths for the upper bound of the *D*_*k*_ interval.

India, Pakistan, Palestine, Ethiopia and China are the only countries represented in the studies that we assessed for data availability that were not included in Karlinsky and Kobak [62]'s analysis. There was evidence of substantial under-reporting of COVID-19 deaths in India [63, 64] while little could be gathered about the reliability of official mortality data for Pakistan, Palestine,^1^ Ethiopia,^2^ and China (but do see [65] and [66]). As such, we excluded the Qutob *et al*. [67] (‘Palestinian population residing in the West Bank’) and He *et al*. [68] (‘Wuhan, China’) studies from the Serotracker-based analysis and excluded the Alemu *et al*. [69] (‘Addis Ababa, Ethiopia’) and the Nisar *et al*. [70] (‘Two neighborhoods of Karachi, Pakistan’) studies from the Chen *et al*.-based analysis. For India, Mukherjee *et al*. [71] and Purkayastha *et al*. [72] estimate UCRs for the entire country as well as for each individual Indian state and union territory. We used these UCRs to adjust the upper bound of the *D_k_* interval for each of the Indian seroprevalence studies (see Supplementary Material for details).

There are two countries represented within our data that were identified by Karlinsky and Kobak [62] as having large discrepancies between the official number of deaths attributed to COVID-19 and the number of excess deaths: Iran (with UCR = 2.4) and Russia (with UCR = 4.5). As such, for Barchuk *et al*. [17] (‘Saint Petersburg, Russia’) and for the Khalagi *et al*. [40] (‘Iran’), we used numbers based on excess deaths for the upper bound of the *D*_*k*_ interval (see *D*_*k*_ numbers in Tables 3 and 4 and see Supplementary Material for details).

Finally, our target of inference is the IFR for the community-dwelling population and does not apply to people living in long-term care (LTC) facilities [also known as ‘nursing homes’ or, in France as ‘Établissement d'hébergement pour personnes âgées dépendantes’ (EHPAD)]. The spread of COVID-19 is substantially different in LTC facilities than in the general population and residents of LTC facilities are particularly vulnerable to severe illness and death from infection; see Danis *et al*. [73]. With this in mind, we made adjustments (when appropriate/possible) to the mortality numbers used in our analysis in order to exclude deaths of LTC residents; see Supplementary Material for details. Modelling the spread and mortality of COVID-19 within LTC facilities will require unique approaches and should be considered in a separate analysis; see the recommendations of Pillemer *et al*. [74].

## Results

The Model as described in the Methods Section, was fit to the two datasets described above. We fit the model using JAGS (just another Gibbs sampler) [75], with five independent chains, each with two million draws (20% burn-in, thinning of 100); see Supplementary Material for details and JAGS code.

**Fig. 3.**
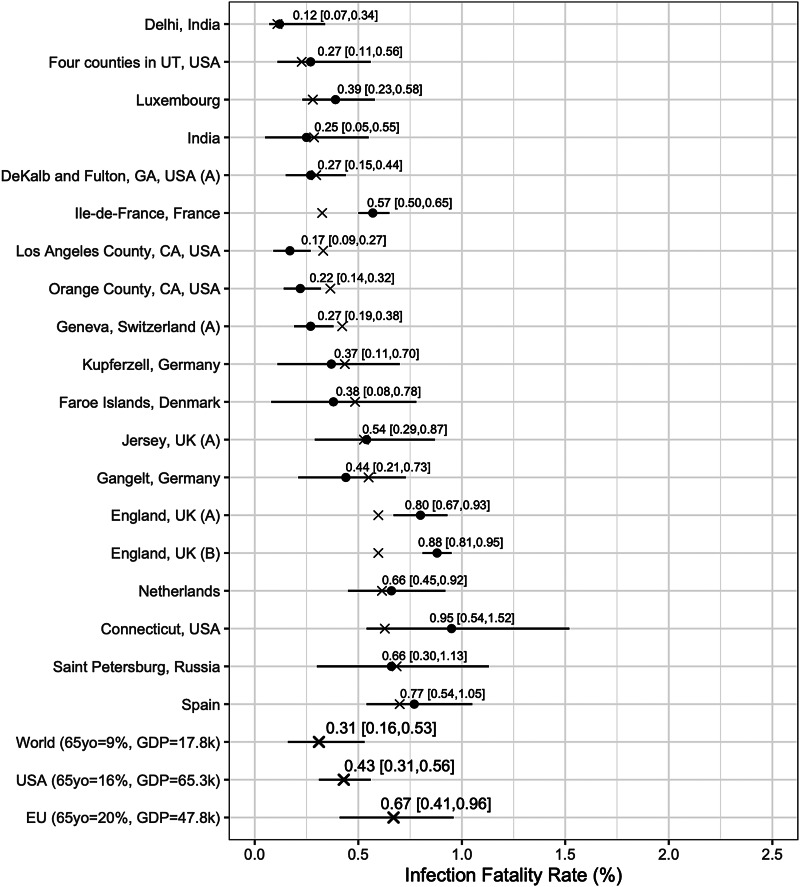
Results from the Chen *et al*.-based analysis: posterior median estimates (black circles) for the *IFR*_*k*_ variables (for *k* = 1, …, 19) with 95% HPD CrIs. Studies are listed from top to bottom in order of increasing fitted values (these values are indicated by ×). Also plotted, under the labels ‘World (65 yo = 9%, GDP = 17.8k)’, ‘USA (65 yo = 16%, GDP = 65.3k)’, ‘EU (65 yo = 20%, GDP = 47.8k)’, are the posterior median estimate and 95% HPD CrIs for the typical IFR corresponding to values for the proportion of the population aged 65 years and older of 9% and for GDP per capita of $ 17 811 (the worldwide values), of 16% and of $ 65 298 (the USA values) and of 20% and of $ 47 828 (the EU values).

**Fig. 4.**
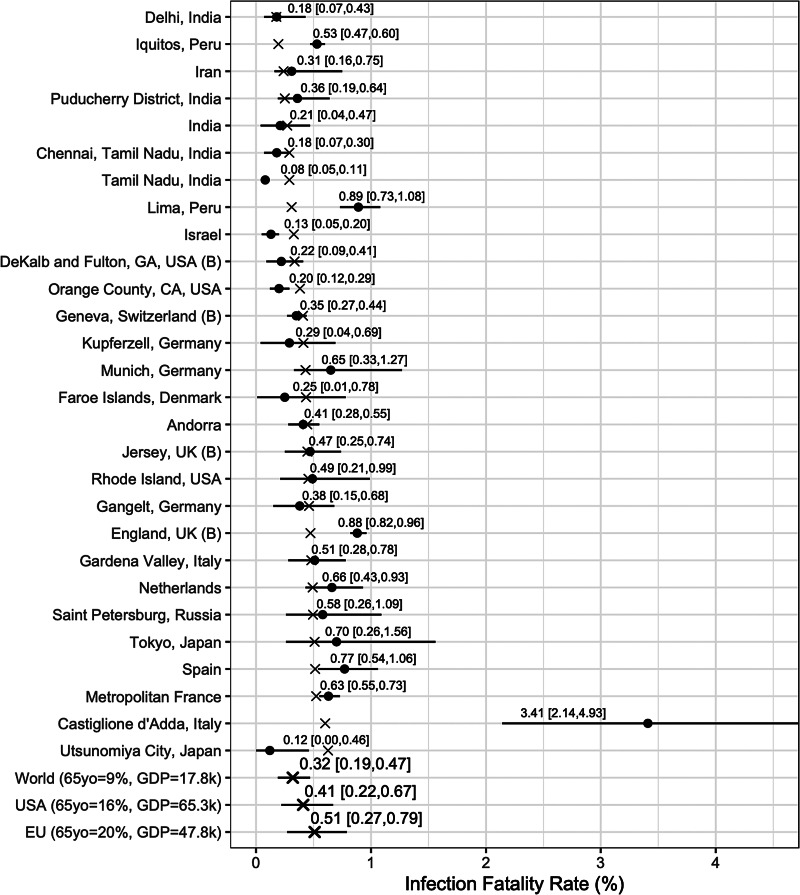
Results from the Serotracker-based analysis: posterior median estimates (black circles) for the *IFR*_*k*_ variables (for *k* = 1, …, 28) with 95% HPD CrIs. Studies are listed from the top to the bottom in order of increasing fitted values (these values are indicated by ×). Also plotted, under the labels ‘World (65 yo = 9%, GDP = 17.8k)’, ‘USA (65 yo = 16%, GDP = 65.3k)’, ‘EU (65 yo = 20%, GDP = 47.8k)’, are the posterior median estimate and 95% HPD CrIs for the typical IFR corresponding to values for the proportion of the population aged 65 years and older of 9% and for GDP per capita of $ 17 811 (the worldwide values), of 16% and of $ 65 298 (the USA values) and of 20% and of $ 47 828 (the EU values).

We report posterior median estimates and 95% highest probability density (HPD) credible intervals (CrI). Figure 3 (for the Chen *et al*.-based analysis) and Figure 4 (for the Serotracker-based analysis) plot the point estimates and CrIs obtained for *IFR*_*k*_, for *k* in 1, …, *K*, respectively; see Supplementary Figures S1 and S2 for *IR*_*k*_ in the Supplementary Material. In these figures, the seroprevalence studies are listed in order of their ‘fitted’ IFR values (the posterior median of *g*^−1^(*θ*_0_ + *θ*_1_*Z*_1*k*_ + *θ*_2_*Z*_2*k*_), for *k* in 1, …, *K*, marked on the plot by the × symbols). Results obtained for the other model parameters are listed in Table 5.

**Table 5.** Parameter estimates (posterior medians and 95% HPD CrIs) obtained from the Chen *et al*.-based analysis and the Serotracker-based analysis

Parameter	Chen *et al*. analysis	Serotracker analysis
*θ*_0_	−5.50, with 95% CrI of (−5.78, −5.22)	−5.56, with 95% CrI of (−5.93, −5.19)
*θ*_1_	0.61, with 95% CrI of (0.21, 1.00)	0.42, with 95% CrI of (−0.40, 1.16)
*θ*_2_	−0.28 with 95% CrI of (−0.67, 0.14)	−0.11, with 95% CrI of (−0.86, 0.69)
*τ*	0.46 with 95% CrI of (0.25, 0.75)	0.84, with 95% CrI of (0.55, 1.23)
*σ*	1.17 with 95% CrI of (0.80, 1.68)	1.53, with 95% CrI of (1.13, 2.05)

In general, the Chen *et al*.-based analysis and the Serotracker-based analysis provide mostly similar results. Notably, the Serotracker-based analysis considers a much more geographically diverse set of seroprevalence studies and several studies that appear to be prominent outliers (e.g. ‘Tamil Nadu’, ‘Castiglione d'Adda, Italy’ and ‘Utsunomiya City, Japan’), see Figure 4. These outliers could be due to infection rates in these populations being markedly different for the elderly relative to the general population.^3^ With regards to heterogeneity, fitting the model without any covariates, one obtains 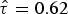 (Chen *et al*.-based analysis) and 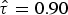 (Serotracker-based analysis). This suggests that the two covariates, 65 yo_*k*_ and GDP_*k*_, account for approximately 45% (=(0.62^2^ − 0.46^2^)/(0.62^2^); Chen *et al*.-based analysis) and 13% (=(0.90^2^ − 0.84^2^)/(0.90^2^); Serotracker-based analysis) of the heterogeneity in the IFR.^4^

Our estimates of 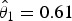 (Chen *et al*.-based analysis) and 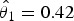 (Serotracker-based analysis) suggest that older populations are more likely to have higher IFRs. This is as expected since age is known to be a very important risk factor [77, 78]. Our estimate of 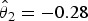 (Chen *et al*.-based analysis) and 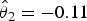 (Serotracker-based analysis) suggest that wealthier populations may be more likely to have lower IFRs. However, the wide CrIs obtained for the *θ*_2_ parameter (in both analyses) suggest a much less definitive conclusion. There are several reasons which might explain this result. As with any observational data analysis, the estimate of *θ*_2_ may suffer from bias due to unobserved confounding and statistical power may be compromised by the presence of outliers and insufficient heterogeneity in the GDP per capita metric across the different populations included in our analyses.

We can infer (by determining the posterior median of *g*^−1^(*θ*_0_ + *θ*_1_*z*_1*_ + *θ*_2_*z*_2*_), for selected values of *z*_1*_ and *z*_2*_) the *typical* IFR amongst populations (be they included in our study or not) having a given proportion of the populace aged over 65 and a given GDP per capita. Thus we calculate posterior point and interval estimates corresponding to age and wealth values that match the population of the entire world (World), the United States (USA) and the European Union (EU) [as listed by the World Bank's World Development Indicators (WDI)]; see ‘World’, ‘USA’ and ‘EU’ rows in Figures 3 and 4. For 65yo = 9% and GDP = $ 17 811, the approximate worldwide values, we obtain, from the Chen *et al*.-based analysis, an across-population average IFR estimate of 0.31%, with a 95% CrI of (0.16%, 0.53%). With the Serotracker-based analysis, we obtain a similar estimate of 0.32%, with a 95% CrI of (0.19%, 0.47%). For 65yo = 16% and GDP = $ 65 298, the USA values, we obtain across-population average IFR estimates of 0.43%, with a 95% CrI of (0.31%, 0.56%) (Chen *et al*.-based analysis) and of 0.41%, with a 95% CrI of (0.22%, 0.67%) (Serotracker-based analysis). Finally, for 65yo = 20% and GDP = $ 47 828, the EU values, we obtain across-population average IFR estimates 0.67%, with a 95% CrI of (0.41%, 0.96%) (Chen *et al*.-based analysis) and of 0.51%, with a 95% CrI of (0.27%, 0.79%) (Serotracker-based analysis). Note that for the ‘World’ predictions, the Serotracker-based analysis has the more precise estimates, while the Chen *et al*.-based estimates are more precise for the ‘USA’ predictions. This is likely due to the fact that the Serotracker-based analysis considers several younger and less wealthy populations, whereas the Chen *et al*.-based analysis considers fewer outliers.

While the infection-rate estimates obtained from the seroprevalence studies should be relatively reliable (due to having satisfied the risk of bias assessments of either Chen *et al*. [9] or Arora *et al*. [10]), the mortality data we collected may be less reliable depending on the target population and source. The data which were not obtained from official and reliable sources may be particularly suspect. With this in mind, as a sensitivity analysis, we repeated both analyses with these data excluded; see results in Supplementary Figures S3 and S4 in the Supplementary Material. Without the excluded studies, we are unable to provide a reasonable ‘World’ estimate (see the extremely wide CrIs). However, the ‘USA’ and ‘EU’ estimates are relatively similar. We also repeated the two analyses using a different set of priors to verify that our results were not overly sensitive to our particular choice of priors. The results of this alternative analysis are very similar to the results of our original analyses; see Supplementary Figures S5 and S6 in the Supplementary Material.

Our estimates are somewhat similar to those obtained in other analyses. Brazeau *et al*. [4], using data from 10 representative seroprevalence studies (identified after screening 175 studies), infer ‘the overall IFR in a typical low-income country, with a population structure, skewed towards younger individuals, to be 0.23% (0.14–0.42% 95% prediction interval range).’ For a ‘typical high-income country, with a greater concentration of elderly individuals,’ Brazeau *et al*. [4] obtain an estimate of 1.15% (95% prediction interval of 0.78–1.79%). Ioannidis [2], using data from seroprevalence studies with sample sizes greater than 500 (and including deaths of LTC residents), obtains a ‘median IFR across all 51 locations’ of 0.27% and (and of 0.23% following an ad-hoc correction to take into account ‘that only one or two types of antibodies’ may have been tested in some seroprevalence studies). Levin *et al*. [3], who restricted their analysis to populations in ‘advanced economies’ (and included deaths of LTC residents) provide age-group specific estimates and country-specific estimates. For instance, for the 45–54 year old age group, Levin *et al*. [3] estimate the IFR to be 0.23% (95% CI of 0.20–0.26%) and for the 55–64 year old age group, 0.75% (95% CI of 0.66–0.87%). For Spain, Levin *et al*. [3] estimate an IFR of 1.90%. For comparison, we estimate the IFR for the community-dwelling population (i.e. excluding deaths of LTC residents) of Spain to be 0.77% (in both analyses). This is similar to the 0.83% estimate obtained by Pastor-Barriuso *et al*. [79] and the 0.75% estimate obtained by Brazeau *et al*. [4] (both of these excluding deaths of LTC residents).

Specifically, with regards to the United States, Sullivan *et al*. [80] estimate the IFR for adults to be 0.85% (95% CrI of 0.76–0.97%) based on a US nationwide seroprevalence survey conducted between August and December, 2020.^5^ Pei *et al*. [81] using a rather complex Bayesian ‘metapopulation’ model conclude that, for the United States during 2020, the IFR likely ‘decreased from around 1% in March to about 0.25% in December.’ For comparison, our ‘USA’ predictions of 0.43% and of 0.41% are based on data obtained mostly between April, 2020 and August, 2020 (see dates in Tables 1 and 2).

## Conclusion

Estimation of the IFR can be incredibly challenging due to the fact that it is a ratio of numbers where both the numerator and the denominator are subject to a wide range of biases. Our proposed method seeks to address some of these biases in a straightforward manner. In our analysis, proper handling of the various sources of uncertainty was a primary focus [82].

With regards to the numerator, we considered the number of deaths as interval-censored data so as to account for the uncertainty in selecting the most relevant number of deaths. While we consider this an improvement over other methods that use a single fixed number, we acknowledge that the specific choice of a 14-day offset is somewhat arbitrary and that the data for deaths also suffer from other sources of bias. Ioannidis [8] notes that the time between infection and death may vary substantially ‘and may be shorter in developing countries where fewer people are long-sustained by medical support.’ In addition to official numbers, we used mortality data based on ‘excess deaths’ statistics for Russia and Iran, since official mortality statistics appeared to be potentially highly inaccurate. We also used adjusted mortality numbers for India based on the best available information. These adjustments are certainly not perfect and we note that ‘excess deaths’ statistics may also suffer from substantial inaccuracies [83].

With regards to the denominator, we looked to data from ‘high-quality’ seroprevalence studies in an effort to avoid biased estimates. However, these data are also not perfect. Seroprevalence studies are severely limited by the representativeness of the individuals they test. Certain groups of individuals who may have very high infection rates are unlikely to be tested in a seroprevalence study (e.g. homeless people). On the other hand, those individuals who have reason to believe they may have been infected, may be more likely to volunteer to participate in a seroprevalence study [58]. It is also likely that seroreversion (loss of detectable antibodies over time) may lead to a seroprevalence study underestimating the true number of infections if the time between the main outbreak and the subsequent antibody testing is substantial [84]. Notably, Axfors and Ioannidis [85] employ a ‘X-fold’-based correction factor to adjust seroprevalence estimates for this type of bias.

The need to improve the quality and reporting of seroprevalence studies cannot be overemphasised.^6^ A major limitation of evidence synthesis is often summarised by the expression ‘garbage in, garbage out’ [86], meaning that if one includes biased studies in one's analysis, the analysis results will themselves be biased [87]. In our two analyses, we only included data from 19 and 28 out of potentially hundreds of seroprevalence studies due primarily to the fact that so few studies were considered reliable and at low risk of bias. Excluding low-quality/biased studies from our analysis was necessary, at least to a certain degree, in order to obtain valid estimates. However, as a consequence of our strict exclusion criteria, much of the world's population is severely under-represented in our data. In related work, Levin *et al*. [88] review the available literature and ‘informally assess studies for risk of bias’ in an attempt to estimate the COVID-19 IFR specifically for developing countries. If the quality of studies were to be correlated with unmeasured factors that impact the IFR, excluding studies based on their perceived quality could lead to unmeasured confounding at a meta-analytic level [89]. Novel methods which allow evidence syntheses to appropriately incorporate biased data are urgently needed. Recently, Campbell *et al*. [90] proposed a partially identified model to combine seroprevalence study data with biased data from official statistics.

Outside of biased data, perhaps the foremost challenge in evidence synthesis using observational data is that necessarily one is forced to make an array of design choices around inclusion/exclusion criteria, statistical modelling approaches and prior specifications [11]. With the two separate analyses and the various additional sensitivity analyses, we were quite encouraged by the stability of our results to perturbations of these inputs.

Reducing the uncertainty around the severity of COVID-19 was of great importance to policy makers and the public during the early stages of the pandemic [91–93] and immense efforts have been made in the collection and analysis of data (e.g. Williamson *et al*. [77]). And yet, even after more than a year, there is still a large amount of uncertainty and unexplained heterogeneity surrounding the COVID-19 IFR, particularly with respect to populations in less affluent countries. While a certain amount of heterogeneity is to be expected [94], identifying factors associated with higher IFRs is the ultimate goal and investigating potential variables that can account for the observed heterogeneity may lead to important insights [89, 95].

We prioritised simplicity in our modelling so as to promote transparency in our findings and to facilitate adaptations to similar, but not identical, data structures. While ‘simple’ is a relative term, note that the entire dataset used for our analyses fits on a single page (in Tables 3 and 4) and that the entire JAGS MCMC code fits on less than a single page (see Supplementary Material). One model extension that could be pursued would involve age stratification of IFR.

Including age-stratification in the model could represent a substantial improvement given that infection in some populations is far from homogeneous (e.g. about 95% of Singapore's COVID-19 infections were among young migrant workers (as of September 2020), which explains the incredibly low case fatality rate [96]). If a factor, such as age, impacts both the risk of infection and the risk of death given infection, then estimating the IFR as we have done in our analysis could be subject to confounding [97]. Age-group specific seroprevalence/mortality data is available for certain geographic areas [98] (although not always consistently reported) and such data could inform an extended version of our model, thereby offering an alternative to the approach described by Levin *et al*. [3] for estimating age-group specific IFRs.

Finally, we must emphasise that the IFR is a moving target. As the pandemic changes, so to does the IFR. Our estimates are based on data from 2020, most of which were obtained more than a year ago. It is likely that, with continual viral mutation of SARS-CoV-2, advances in treatment and the availability of vaccines, the current IFR in many places is now markedly different than it was earlier in 2020 [81, 99, 100].

## Key messages

- The COVID-19 IFR is estimated to be about 0.32% for a typical community-dwelling population where 9% of individuals are over 65 years old and where the GDP per capita is $17.8k (the approximate worldwide averages). For a typical community-dwelling population with the age and wealth of the United States we estimate the IFR to be approximately 0.42%.
- Any estimation of the COVID-19 IFR should take into account the various uncertainties and potential biases in both the mortality data and the seroprevalence data.
- Bayesian methods with interval censoring are well suited for complex evidence synthesis tasks such as estimating the COVID-19 IFR.

## Data Availability

Data and code used for the analysis are available in the Supplementary Material and at the OSF project ‘Inferring the COVID-19 IFR in the community-dwelling population: a simple Bayesian evidence synthesis of seroprevalence study data and imprecise mortality data’ (DOI 10.17605/OSF.IO/34SQ5); see osf.io/34sq5.

## Footnotes

1 Official regional death numbers for Palestine are available from the Palestinian government dashboard (see https://corona.ps/details; accessed July 28, 2021).

2 Official regional death numbers for Ethiopia have been made available previously (e.g., http://web.archive.org/web/2020*/https://www.covid19.et/covid-19/ and the Twitter account: https://twitter.com/Harun_Asefa/status/1259069832877793280; accessed August 4, 2021).

3 Malani et al. [41] (‘Tamil Nadu’) note that: ‘Seroprevalence among the elderly (70+: 25.8%) is significantly lower than among the working age populations (age 40-49: 31.6%; *p* < 0.001) or the young (18-29: 30.7%; *p* < 0.001).’ Pagani et al. [45] (‘Castiglione d'Adda, Italy’) also report that the elderly are more likely to have been infected (‘strong association observed between IgG seroprevalence and age’). On the other hand, note that among the 181 participants in the Nawa et al. [44] study (‘Utsunomiya City, Japan’) who were aged 65 years or older, none were positive. This suggests that infection in the elderly may be lower than in the general population. However, since the Nawa et al. [44] found only 3 positive cases out of a total of 742 individuals tested, inference on this is limited.

4 For reference, Levin et al. [3] conclude that 87% of the heterogeneity in the IFR (of advanced economies) can be explained by variations in age composition and age-specific prevalence of COVID-19. However, note that the linear regression analysis used to obtain this 87% result is done without an intercept term (see Levin et al. [3] - Figure 6). A linear regression with intercept results in a value of 43%. The intraclass correlation coefficient (ICC) [76] between Levin et al. [3]'s predicted IFRs and the observed IFRs is 0.65 (after removing one outlier, ‘Portugal’), suggesting that the extent of agreement is reasonably high but nowhere near perfect.

5 While Sullivan et al. [80] used a nationwide representative sampling frame, the results may be subject to sizable selection bias given the low response rate of 12.6%.

6 Bobrovitz et al. [53] conclude that a majority of COVID-19 seroprevalence studies are ‘at high risk of bias […], often for not statistically correcting for demographics or for test sensitivity and specificity, using non-probability sampling methods, and using non-representative sample frames.’ Shook-Sa et al. [58] note that ‘it is very difficult to estimate the level of bias introduced by convenience sampling.’

## Supplementary material

For supplementary material accompanying this paper visit http://dx.doi.org/10.1017/S0950268821002405. click here to view supplementary material
