# Supplementary material for: Inferring the COVID-19 infection fatality rate in the community-dwelling population: a simple Bayesian evidence synthesis of seroprevalence study data and imprecise mortality data
Source: Epidemiol Infect. 2021 Nov 8;149:e243. doi: 10.1017/S0950268821002405 (PMC8632419; doi:10.1017/S0950268821002405)
Supplement: Supplementary file 1 [file hygsup.zip › S0950268821002405sup004.pdf]

Inferring the COVID-19 infection fatality rate in the community-dwelling population: a simple Bayesian evidence synthesis of seroprevalence study data and imprecise mortality data

Harlan Campbell

Department of Statistics, University of British Columbia
Vancouver, British Columbia, Canada
harlan.campbell@stat.ubc.ca
ORCID ID: 0000-0002-0959-1594

Paul Gustafson
Department of Statistics, University of British Columbia
Vancouver, British Columbia, Canada
ORCID ID: 0000-0002-2375-5006

November 2, 2021

S1 Supplementary Material

S1.1 Excluded studies - Chen et al.-based analysis

Figure 1 shows a flowchart of the literature search and Table S1 lists all the excluded studies. Among the 38 articles obtained from the Chen et al. review, four studies represented results from different phases of the same study. For each of these we considered only the data from the earliest phase of the study. Stringhini et al. [90] and Richard et al. [73] are two publications that report the earlier and later phases, respectively, of the same study of Geneva, Switzerland. We considered only data from the earlier phase as reported in Stringhini et al. [90]. Murhekar et al. [52] and Murhekar et al. [53] are two publications that report the earlier and later phases, respectively, of the same study in India. We considered only data from the first phase as reported in [52].

Note that, while similar in many ways, Ward et al. [100] and Office of National Statistics [57] are two different large-sample studies. The Ward et al. [100] study is based on the "REACT-2" survey which is led by Imperial College London, while the Office of National Statistics [57] study is based on the "Coronavirus (COVID-19) Infection Survey" which is conducted by a partnership between the University of Oxford, University of Manchester, Public Health England and Wellcome Trust. Spiers [85] discusses the differences between the two surveys.

We excluded eight studies that used "non-probability" or "convenience" sampling methods (e.g, studies in which participants were recruited using social media, or recruited in shopping centers). Specifically, we excluded: (1) McLaughlin et al. [47] who sampled individuals from a list of volunteers (Arora et al. [6] classifies the sampling method for this study as "self-referral"); (2) Rosenberg et al. [74] who sampled individuals at grocery stores ("convenience" [6]); (3) Appa et al. [5] who recruited volunteers with support from Bolinas community leaders ("self-referral" [6]); (4) Bendavid et al. [11] who recruited participants by placing targeted advertisements on Facebook ("stratified non-probability" [6]); (5) Gudbjartsson et al. [27] ("convenience" [6]); (6) Borges et al. [14] ("convenience" [6]); (7) Ling et al. [40] ("self-referral" [6]); and (8) Naranbhai et al. [54] ("convenience" [6]). Due to new information about the sensitivity of the Wondfo antibody tests (Silveira et al. [82]: "Our findings cast serious doubts about the use of this brand of rapid tests for epidemiological studies."), we excluded the Hallal et al. [28] Brazil study (but do see: Marra and Quartin [46]). One study (Malani et al. [45]) was excluded due to a narrowly defined target population.

Two additional studies were not included because the articles failed to report 95% uncertainty intervals for the estimated infection rate in the target population [44, 99] and five additional studies were not included due to unavailable reliable mortality data for the specific target populations [1, 56, 64, 92, 79].

**Author**	**Location**	**Reason for exclusion**
Alemu et al. [1]	Addis Ababa, Ethiopia	Not included in Karlinsky and Kobak [33] analysis.
Appa et al. [5]	Bolinas, CA, USA	Convenience sampling (recruited volunteers with support from Bolinas community leaders ("self-referral" [6])).
Bendavid et al. [11]	Santa Clara county, CA, USA	Recruited participants by placing targeted advertisements on Facebook ("stratified non-probability"[6]).

Borges et al. [14]	Sergipe (10 cities), Brazil	Convenience sampling ("convenience" [6]) (but also: the 95% uncertainty interval not provided for the prevalence estimate).
Gudbjartsson et al. [27]	Iceland	Convenience sampling ("convenience" [6]).
Hallal et al. [28]	Brazil (83 cities)	Excluded due to new information about the sensitivity of the Wondfo antibody test [82].
Ling et al. [40]	Wuhan, China	Convenience sampling ("Self-referral" [6]) (but also: death data may be unreliable; see [41] and [93]).
Majiya et al. [44]	Nigeria	95% uncertainty interval not provided for the prevalence estimate.
Malani et al. [45]	Mumbai, India	The seroprevalence study provides two estimates: (1) for those living in the slums of the Matunga, Chembur West, and Dahisar Each wards; and (2) for those living in the non-slums of the Matunga, Chembur West, and Dahisar Each wards. These two target populations are too narrowly defined for available mortality data. Note, however, that some official mortality data appears at the ward level is available; see for example: "Ward-wise breakdown of positive cases" information in `https://web.archive.org/web/20200625110946/http://stopcoronavirus.mcgm.gov.in/assets/docs/Dashboard.pdf` (accessed August 4, 2021).

McLaughlin et al. [47]	Blaine, ID, USA	Convenience sampling (sampled individuals from a list of volunteers ("self-referral" Arora et al. [6])).
Murhekar et al. (B) [51]	India	Duplicate ([52] report the results from the same study).
Naranbhai et al. [54]	Chelsea, MA, USA	Convenience sampling ("convenience" [6]) (but also: 95% uncertainty interval not provided for the prevalence estimate).
Nisar et al. [56]	Two neighborhoods of Karachi, Pakistan	Not included in Karlinsky and Kobak [33] analysis.
Poustchi et al. [64]	Iran (18 cities)	Death data not found for the target population.
Richard et al. [73]	Geneva, Switzerland	Duplicate ([90] report results from the same study).
Rosenberg et al. [74]	New York state, USA	Convenience sampling (sampled individuals at grocery stores ("convenience" Arora et al. [6])).
Shakiba et al. [79]	Guilan Province, Iran	Death data not found.
Tess et al. [92]	Six districts of São Paulo, Brazil	Death data not found.
Wang et al. [99]	Beijing, China	95% uncertainty interval not provided for the prevalence estimate.

Table S1: List of excluded studies and reason for exclusion for the Chen et al. - based analysis.

S1.2 Excluded studies - Serotracker-based analysis

Figure 2 shows a flowchart of the literature search and Table S2 lists all the excluded studies. Among the 45 articles obtained from the Serotracker review, six studies represented results from different phases of the same study. For each of these we considered only the data from the earliest phase of the study. For instance, Murhekar et al. [52] and Murhekar et al. [53] report the results from an earlier and a later phase of the same study in India. We only include Murhekar et al. [52] for our analysis. We excluded one additional study that used "convenience" sampling methods [88] and two studies that used the Wondfo antibody test [39, 81] (without adequate adjustment given the recent information about the sensitivity of the Wondfo test Silveira et al. [82]). We excluded two additional studies that had a narrowly defined target population[7, 96].

One additional study was not included because the article failed to report a 95% uncertainty interval for the estimated infection rate in the target population (Truc and Gervino [94] focused on estimating the longitudinal changes in antibodies levels rather than the prevalence rate in Cogne, Italy.). Note that the "Andorra" study [26] also failed to report a 95% uncertainty interval for the estimated infection rate in the target population. However, we chose to exceptionally include the study due to the fact that it represents one of the largest seroprevalence studies conducted: a total of 70,494 inhabitants (90.9% of the population of Andorra) participated in the study. In their published article Royo-Cebrecos et al. [75] explain that 95% confidence intervals are not provided because such intervals "would be extremely narrow and potentially misleading given that they do not account for the potential bias that non-participating individuals could cause on our central seroprevalence estimate." We therefore defined, in order to be very cautious, a very wide (yet entirely arbitrary) 95% confidence interval of [10.5%, 11.5%] around the 11.0% point estimate. Eight additional studies were not included due to unavailable reliable mortality data for the specific target populations.

**Author**	**Location**	**Reason for exclusion**
Backhaus et al. [7]	Dusseldorf, Germany	Sample not representative of overall population: only "young people between the ages of 18-30" and individuals in the "fire brigade and rescue services".
Beaumont et al. [10]	Three neighbourhoods in the city of Perpignan, France	Death data not found for the target population (three specific neighbourhoods (Saint-Jacques, Haut-Vernet, and Nouveau Logis) in the city of Perpignan, France).
Gégout-Petit et al. [23]	Grand Nancy metropolitan area, France	Death data not found; however, note that `coronadatascraper.com` does have data for the larger region of Meurthe-et-Moselle, Grand Est, France.

He et al. [30]	Wuhan, China	Not included in Karlinsky and Kobak [33]'s analysis and see [41] and [93] who discuss issues as to why this is particularly challenging and controversial data to validate.
Huamaní et al. [31]	Cusco, Peru	Death data not found for the target population (”three settings in Cusco: (1) Cusco city, (2) the periphery of Cusco, and (3) Quillabamba city”).
Khan et al. [36]	Vale Kashmir, India	Death data not found (official death numbers can be found on ”Official Twitter handle of Department of Information and Public Relations, Govt of Jammu & Kashmir (“@diprjk”). While not specifically referenced, the numbers reported by this source are consistent with those used in Khan et al. [35].) Mukherjee et al. [50] does not provide an estimated underreporting factor for Kashmir noting that this is “[o]wing to lack of sufficient data”.
Li et al. [39]	Wuhan City, Hubei-ex-Wuhan, and six provinces, China	Excluded due to new information about the sensitivity of the Wondfo antibody test Silveira et al. [82].
Murhekar et al. (B) [53]	India	Duplicate ([52] report the results from the same study).
Pérez-Olmeda et al. [60]	Spain	Duplicate ([63] report the results from the same study).
Poustchi et al. [64]	18 cities of Iran	Death data not found for the target population.
Qutob et al. [67]	Palestinian population residing in the West Bank	Not included in Karlinsky and Kobak [33]'s analysis.

Ramaswamy et al. [70]	Jabalpur Municipal Corporation ("City of Jabalpur")	While official death numbers are available for the District of Jabalpur (from the "Official Account of the Directorate of Health Services, Government of Madhya Pradesh. (e.g., `https://twitter.com/healthminmp/status/1346107670869155843/photo/1`; accessed July 27, 2021)), we could not find data for the City of Jabalpur.
Silveira et al. [81]	Regions of Brazil	Excluded due to new information about the sensitivity of the Wondfo antibody test [82].
Stefanelli et al. [88]	Five municipalities of the Autonomous Province of Trento, Italy	Convenience sampling.
Stringhini et al. (B) [91]	Geneva, Switzerland	Duplicate ([73] report the results from the same study).
Truc et al. [94]	Municipality of Cogne, Italy	95% uncertainty interval not provided for the prevalence estimate.
Ulyte et al. [96]	Zurich, Switzerland	Sample not representative of overall population: only school children.

Table S2: List of excluded studies and reason for exclusion for the Serotracker-based analysis.

S1.3 Details on mortality and covariate data

- For Álvarez-Antonio et al. [2] ("Iquitos, Peru") (see also Álvarez-Antonio et al. [3]), we used official (post-audit) numbers from the Peru Ministry of Health (MINSA) (see `https://www.datosabiertos.gob.pe/dataset/fallecidos-por-covid-19-ministerio-de-salud-minsa/resource/4b7636f3-5f0c-4404-8526`) for mortality numbers. We included deaths recorded for 4 districts ("SAN JUAN BAUTISTA", "BELEN", "PUNCHANA", and "IQUITOS") in the Loreto department. Note that recording the accurate number of deaths is perhaps particularly challenging in Iquitos; see Fraser [22]. In order to acknowledge

that there is substantial uncertainty in the post-audit mortality numbers, we widened the interval by lowering the lower bound by 10% and increasing the upper bound by 10%. Limited information is available about mortality amongst LTC residents [98]. As such, no LTC-adjustments were made to the mortality numbers. Data for the proportion aged over 65 is from a 2004 World Health Organization report (see https://www.who.int/ageing/projects/intra/phase_two/alc_intra2_cp_peru.pdf; accessed August 2, 2021). GDP data is from the "USD per head, current prices, current PPP" value listed by the Organisation for Economic Co-operation and Development's Gross Domestic Product, Large regions TL2 database ("OECD database"; see https://stats.oecd.org/, accessed August 10, 2021) for the Loreto region (PE16) . Population data is from the published article [3].

- For Warszawski et al. [101] ("Metropolitan, France"), we obtained mortality data from the French government COVID-19 dashboard (see https://dashboard.covid19.data.gouv.fr/vue-d-ensemble?location=FRA; accessed on August 4, 2021), summing up the cumulative deaths for the 13 regions of Metropolitan France (i.e., European France). (235+394+1616+407+471+6710+3256+959+371+1562+469+836+56 and 257+427+1804+461+528+7358+3518+1032+411+1721+508+927+59). Note that this death total does not include deaths that occurred in LTC facilities. These are listed separately on the French government COVID-19 dashboard under "EHPAD et EMS". Data for the proportion aged over 65 is for France from 2019 as listed by World Bank's World Development Indicators (WDI) data (https://data.worldbank.org/indicator/SP.POP.65UP.TO.ZS?locations=FR; accessed August 4, 2021). GDP data is from the OECD database. Population data is from INSEE in report titled "Population totale par sexe et âge au 1er janvier 2020, France métropolitaine" (see https://www.insee.fr/fr/statistiques/1892088?sommaire=1912926; accessed August 4, 2021).

- For Bajema et al. [8] ("DeKalb and Fulton, GA, USA (B)"), we obtained the number of deaths for DeKalb, and Fulton counties from the county-level COVID-19 dataset curated by the New York Times available at: github.com/nytimes/covid-19-data (accessed on April 28, 2021). According to the New York Times analysis, (see: https://web.archive.org/web/20200627101803/https://www.nytimes.com/interactive/2020/us/coronavirus-nursing-homes.html) about 45% of COVID-19 deaths in the state of Georgia were among nursing home residents (as of June 27, 2020). We therefore adjusted the numbers by 45% and obtained 122 (=221-0.45*221) and 136 (=247-0.45*247). Data for the proportion aged over 65 is from https://github.com/JieYingWu/COVID-19_US_County-level_Summaries/blob/master/data/README.md (accessed on April 29, 2021). GDP value is from value listed

in the OECD database for the state of Georgia, USA (US13). Population is from the United States census (https://www.census.gov/quickfacts/fultoncountygeorgia and https://www.census.gov/quickfacts/dekalbcountygeorgia ; accessed July 28, 2021) (i.e., 759297 + 1063937). Note that both Bajema et al. [8] and Biggs et al. [13] report the results from the same seroprevalence study.

- For Barchuk et al. [9] ("Saint Petersburg, Russia"), we used excess death numbers as reported by Kobak [37] for the upper bound and official government numbers for the lower bound. Russian official government statistics appear to underestimate the true number of fatalities by a substantial factor [33]. No information was found for mortality amongst LTC residents in Russia; however see: https://www.hrw.org/news/2020/06/02/russia-publish-data-about-covid-19-institutional-care. Data for the proportion aged over 65 is from Barchuk et al. [9] (see Table A3 "KOUZh-2018"). GDP value is from OECD database for the "Federal City of Saint Petersburg" (RU29) region. The population value is from https://en.wikipedia.org/wiki/Saint_Petersburg (accessed June 21, 2021)).

- For Biggs et al. [13] ("DeKalb and Fulton, GA, USA (A)"), the number of deaths for DeKalb, and Fulton counties was obtained from the county-level COVID-19 dataset curated by the New York Times available at: github.com/nytimes/covid-19-data (accessed on April 28, 2021). Data for the proportion aged over 65 is from https://github.com/JieYingWu/COVID-19_US_County-level_Summaries/blob/master/data/README.md (accessed on April 29, 2021). According to the New York Times analysis, (see: https://web.archive.org/web/20200627101803/https://www.nytimes.com/interactive/2020/us/coronavirus-nursing-homes.html; accessed Oct. 12, 2021) about 45% of COVID-19 deaths in the state of Georgia were among nursing home residents (as of June 27, 2020). We adjusted the numbers by 45% and obtained 122 (=221-0.45*221) and 136 (=247-0.45*247). Data for the proportion aged over 65 is from https://github.com/JieYingWu/COVID-19_US_County-level_Summaries/blob/master/data/README.md (accessed on April 29, 2021). GDP value is from value listed in the OECD database for the state of Georgia, USA (US13). Population is from the United States census (https://www.census.gov/quickfacts/fultoncountygeorgia and https://www.census.gov/quickfacts/dekalbcountygeorgia; accessed July 28, 2021) (i.e., 759297 + 1063937). Note that both Bajema et al. [8] and Biggs et al. [13] report the results from the same seroprevalence study.

- For Bruckner et al. [15] ("Orange County, CA, USA"), we obtained the number of cumulative deaths for Orange County from the Orange County Public Works (as referenced by Bruckner et al. [15]) at: data-ocpw.opendata.arcgis.com/datasets/

2ec9342ffc814cf58161b1cca57365fd_0 (accessed on April 28, 2021). Orange County Public Works reports deaths of skilled nursing facility (SNF) residents separately. For 2020-07-24, there were a total of 759 deaths including 247 deaths of SNF residents. For 2020-08-30, there were a total of 1209 deaths including 370 deaths of SNF residents. We recorded mortality numbers accordingly. Data for the proportion aged over 65 is from https://github.com/JieYingWu/COVID-19_US_County-level_Summaries/blob/master/data/README.md (accessed on April 29, 2021). GDP value is from value listed for the state of California, USA (US06) from the OECD database. Population value obtained from the United States census (https://www.census.gov/quickfacts/orangecountycalifornia; accessed June 27, 2021).

- For Carrat et al. [17] ("Île-de-France, France"), we only consider Île-de-France phase of the study (see Supplementary Table 1 in Carrat et al. [17] for sampling dates). Data for the number of deaths for Île-de-France was obtained from the French government COVID-19 dashboard (see https://dashboard.covid19.data.gouv.fr/vue-d-ensemble?location=FRA; accessed on August 4, 2021). Note that this death total does not include deaths that occurred in long term care facilities ("EHPAD et EMS"). Data for the proportion aged over 65 is from https://contrevues.paris/ile-de-france-comment-le-vieillissement-de-la-population-impacte-lhabitat/ (accessed on April 29, 2021). GDP value is from value listed for Île-de-France region (FR1) from OECD database. Population data from the Institut national de la statistique et des etudes economiques (INSEE) (see https://www.insee.fr/fr/statistiques/5002478; accessed July 29, 2021).

- For Chan et al. [18] ("Rhode Island, USA"), we obtained mortality data from the Rhode Island Department of Health available at https://ri-department-of-health-covid-19-data-rihealth.hub.arcgis.com/ (accessed July 28, 2021). Note that Chan et al. [18] state that: "As of May 31, Rhode Island had reported 827 cumulative lab-confirmed SARS- CoV-2–involved deaths; 78.5% of these deaths were associated with congregate care facilities. We therefore reduced the mortality numbers by 78.5% ($= 684 \times (1-0.785)$ and $= 877 \times (1-0.785)$). Data for the proportion aged over 65 is from the U.S. Department of Commerce https://www.census.gov/quickfacts/RI (accessed on June 21, 2021). GDP value is from value listed for the state of Rhode Island, USA (US44) in the OECD database. The population value is obtained from the United States census (https://www.census.gov/quickfacts/RI; accessed July 28, 2021).

- For Govern d'Andorra [26] ("Andorra") (see also Royo-Cebrecos et al. [75]), we obtained mortality numbers from the Government of Andorra (https://www.govern.ad/coronavirus; accessed on June 28, 2021) as tabulated by Wikipedia (https://en.

wikipedia.org/wiki/COVID-19_pandemic_in_Andorra; accessed on June 28, 2021). We substracted 31.45% of deaths (35 = 51-0.3145*51 for both lower and upper bounds) following the reporting of Carrasco et al. [16] which found that 39 out of 124 COVID-19 deaths in Andorra (for the period between March, 2020 and April, 2021) were amongst residents in nursing homes. Carrasco et al. [16] note that: "218/354 (61.6%) residents in nursing homes were infected and the fatality rate was 39/218 (17.8%)." Data for the proportion aged over 65 is from https://www.indexmundi.com/andorra/demographics_profile.html (accessed on June 28, 2021) and GDP data is from The World Factbook (https://statisticstimes.com/economy/countries-by-gdp-capita-ppp.php; accessed on June 28, 2021)). The population number is as stated in the Royo-Cebrecos et al. [75] which is the published article reporting on the study.

- For Kar et al. [32] ("Puducherry District, India"), data for the number of deaths for the Puducherry District was obtained from https://covid19dashboard.py.gov.in/Reporting/District (accessed on August 5, 2021). We multiplied the number recorded for 14 days after the end of the sampling window by a factor of 2.1 (based on the upper bound of Mukherjee et al. [50]'s estimated underreporting factor for Puducherry) in order to account for potential underreporting. As such, our interval is relatively wide and reflects the substantial uncertainty in the true number of deaths. There is insufficient information about deaths of LTC residents in India to make any LTC adjustments; see Rajagopalan et al. [69]. Data for the proportion aged over 65 is from http://statisticstimes.com/demographics/india/puducherry-population.php (accessed on June 22, 2021). GDP value is from value listed for Puducherry (IN34) from OECD database. Population for Puducherry District from Kar et al. [32] who note that: "Puducherry district, population $\approx$ 1.25 million, is located in southern India."

- For Khalagi et al. [34] ("Iran"), Karlinsky and Kobak [33] suggest that official mortality data for Iran is highly inaccurate and suggest that there have been approx. 58,092 excess deaths in Iran for the period until Sept. 21, 2020. This coincides with the estimate provided by ghafari2020excess of 58,900 (95% CI 46,900 - 69,500) for the period from December 22, 2019 to September 21, 2020. Excess mortality data is only available in quarterly format from Kobak [37]. Therefore it is not possible to obtain mortality data for the specific dates we require: 2020-08-17 and 2020-11-14. Official statistics for these dates are 19,804 and 41,034 (see https://en.wikipedia.org/wiki/COVID-19_pandemic_in_Iran; accessed on June 28, 2021, which cites the Iranian Ministry of Health and Medical Education). We used 19,804 for the lower bound and multiplied 41,034 by Kobak [37]'s estimated undercount ratio of 2.4 to obtain an upper bound of 98071. (see https://www.ncbi.nlm.nih.gov/pmc/articles/PMC7852240/pdf/nihpp-2021.01.27.21250604v3.pdf; ac-

cessed on June 28, 2021). In Iran, LTC is provided mostly informally by family caregivers [24]. As such, no adjustments were made to exclude deaths of LTC residents. Data for the population and the proportion of the population aged over 65 is from the World Bank World Development Indicators (WDI) data (https://data.worldbank.org/indicator/SP.POP.65UP.TO.ZS?locations=IR; accessed on June 28, 2021). GDP value is also from the WDI data (see: https://data.worldbank.org/indicator/NY.GDP.PCAP.PP.CD (last updated 2021-04-26)).

- For Mahajan et al. [43] ("Connecticut, USA"), mortality data obtained from Wikipedia (https://en.wikipedia.org/wiki/COVID-19_pandemic_in_Connecticut; accessed on July 22, 2021), who reference (https://portal.ct.gov/Coronavirus). Mortality numbers (4,287 and 4,450) are multiplied by 26.5% (i.e. by 1079/4071) due to the fact that approx. 26.5% of COVID-19 deaths in Connecticut occurred in the community-dwelling population (i.e., "non-congregate" population) according to Mahajan et al. [42]. Data for the proportion aged over 65 is derived from Table 1 of Mahajan et al. [42]. GDP value is from value listed for the state of Connecticut, USA (US09) from the OECD database. Population value is obtained from [42].

- For Malani et al. [45] ("Tamil Nadu, India"), the population of Tamil-Nadu, India, was obtained from https://www.indiacensus.net/states/tamil-nadu (accessed July 27, 2021). Mortality data for the number of deaths for Tamil Nadu was obtained from the Tamil Nadu Government (see https://stopcorona.tn.gov.in/archive/; accessed June 24, 2021). We multiplied the number recorded for 14 days after the end of the sampling window of 11,909 by a factor of 2.5 (based on the upper bound of Mukherjee et al. [50]'s estimated underreporting factor for Tamil Nadu). There is insufficient information about deaths of LTC residents in India to make any LTC adjustments; see Rajagopalan et al. [69]. GDP value is from value listed for Tamil-Nadu (IN33) from the OECD database. Data for the proportion aged over 65 is from https://statisticstimes.com/demographics/india/tamil-nadu-population.php (accessed July 27, 2021).

- For Melotti et al. [48] ("Gardena Valley, Italy"), we obtained information from the report "Study on the prevalence of Covid-19 in the Val Gardena - June 2020" (see: https://astat.provincia.bz.it/it/news-pubblicazioni-info.asp?news_action=300&news_image_id=1074603; accessed July 27, 2021). In this report, it is noted that "the number of deaths from this disease in 17 people...". Stefano Lombardo (in a personal communication) notes that the source of this number is Astat, Bolzano ("statistics on deaths by cause") and has issued a correction (via personal communication) to indicate that the number should be 20 ("In spring 2020 the covid19-deaths in Gardena were estimated at 17; at the end of the year the official statistics closed at

20 (march+april).") In a further personal communication (Oct. 18, 2021), Lombardo clarified that out of these 20 deaths 3 occurred at home, 12 at the hospital, and 5 at a nursing home. The study looks specifically at three municipalities (Ortisei, Santa Cristina, and Selva) and we used age data from `https://www.citypopulation.de/en/italy/trentinoaltoadige/bolzano/021089__selva_di_val_gardena/` and `http://italia.indettaglio.it/eng/trentinoaltoadige/ortisei.html` (accessed August 4, 2021). GDP value is from OECD database for the Province of Bolzano-Bozen (ITH1).

- For Ministry of Health of Israel [49] ("Israel") (see also Reicher et al. [71]), mortality data obtained from `https://en.wikipedia.org/wiki/COVID-19_pandemic_in_Israel` (accessed July 27, 2021) which references Israel's Ministry of Health' official coronavirus updates, it's Telegram channel, and Israel's Corona National Information and Knowledge Center. According to Tsadok-Rosenbluth et al. [95], a large porpotion of COVID-19 deaths in Israel occurred in long-term care facilities (LTCFs). Specifically, by the end of July, 2020 (2020/07/28), the percentage of deaths that occurred in LTCFs was 51%, and by the end of September, 2020 (2020/09/29), the percentage of deaths that occurred in LTCFs was 40%. Based on these figures we adjusted the death numbers 382 and 1659 to: 187 (=382-0.51*382) and 995 (=1659-0.4*1659). Data for the proportion aged over 65 is from `https://brookdale.jdc.org.il/wp-content/uploads/2018/02/MJB-Facts_and_Figures_Elderly-65_in_Israel-2018_English.pdf` (accessed July 27, 2021) and GDP value is from OECD. Population for Israel is from World Bank's World Development Indicators (WDI) data. Note that while [6] categorizes the sampling method of the Ministry of Health of Israel [49] study as "stratified probability," Reicher et al. [71] note that the study used blood samples from "insured individuals who arrived at the HMOs [Health Maintenance Organizations] to undergo a blood test for any reason" and caution that, as a consequence, the "study might not reliably represent the entire population."

- For Murhekar et al. [52] ("India"), we obtained the number of cumulative deaths for India, from the Our World in Data COVID-19 dataset available at: `ourworldindata.org/coronavirus/country/india` (accessed on April 28, 2021). We multiplied the number recorded for 14 days after the end of the sampling window of 12,573 by a factor of 3.64 (based on the upper bound of Purkayastha et al. [65]'s estimated underreporting factor for India (first wave)) in order to account for potential underreporting. As such, our interval is relatively wide and reflects the uncertainty in the true number of deaths: [4172, 45766]. (Purkayastha et al. [65]: "Estimates from epidemiological models: For wave 1 our estimate ... [is] an underreporting factor for cases estimated at 11.11 (95% CrI 10.71 – 11.47) and for deaths at 3.56 (95% CrI 3.48 – 3.64).") There is insufficient information about

deaths of LTC residents in India to make any LTC adjustments; see Rajagopalan et al. [69]. Data for the proportion aged over 65 is from World Bank's World Development Indicators (WDI) data (see https://data.worldbank.org/indicator/SP.POP.65UP.TO.ZS (last updated 2021-03-19)). GDP value is from OECD database. Population value is from the World Bank (see https://data.worldbank.org/indicator/SP.POP.TOTL?locations=IN; accessed on April 28, 2021).

- For Nawa et al. [55], we concluded that 0 deaths had occurred based on official records for the Tochigi prefecture (see https://www.nippon.com/en/japan-data/h00657/; accessed July 12th, 2021). For the infection interval we use the lowest bound of the confidence interval given for the "unimputed dataset" and the upper bound of the confidence interval given for the "imputed dataset" from Table 1 of Nawa et al. [55]. Note that among the 181 participants in the Nawa et al. [55] study who were aged 65 years or older none were positive (0/181). This suggests that the prevalence amongst those at greatest risk of death (i.e., amongst the elderly) may be lower than in the greater population. However, since the Nawa et al. [55] found only 3 positive cases out of 742 individuals tested, inference on this is limited. Also note that, as of July 13, 2020, amongst the 36 individuals who tested positive by pcr-testing in Utsunomiya City, 4 were in their 70s and none were older than 80 (see https://web.archive.org/web/20200714014725/https://www.city.utsunomiya.tochigi.jp/kurashi/kenko/kansensho/etc/1023506.html). Data for proportion aged over 65 is from https://www8.cao.go.jp/kourei/english/annualreport/2017/pdf/c1-1.pdf (accessed July 12th, 2021). GDP value is from OECD database for Northern-Kanto, Koshin (JPC) region. Population value is from https://www.city.utsunomiya.tochigi.jp/shisei/gaiyo/1007461.html (accessed July 12th).

- For Office of National Statistics [57] ("England, UK (A)"), infection rate numbers were obtained from Table 3a of https://www.ons.gov.uk/file?uri=/peoplepopulationandcommunity/healthandsocialcare/conditionsanddiseases/datasets/coronaviruscovid19infectionsurveydata/2020/previous/v26/covid19infectionsurveydatasets20201002.xlsx; (accessed on April 28, 2021). This dataset specifies that "These statistics refer to infections reported in the community, by which we mean private households. These figures exclude infections reported in hospitals, care homes or other institutional settings." We used mortality numbers obtained from the Office of National Statistics, dataset ("deaths registered weekly in England and Wales"; see publishedweek532020.xlsx, sheet "UK - Covid-19 - Weekly occurrences") retrieved from https://www.ons.gov.uk/peoplepopulationandcommunity/birthsdeathsandmarriages/deaths/datasets/

weeklyprovisionalfiguresondeathsregisteredinenglandandwales (accessed Oct. 12, 2021). We subtracted those deaths attributed to Wales, and subtracted deaths attributed to nursing home residents as recorded in the "Deaths involving COVID-19 in the care sector, England and Wales" dataset (available at https://www.ons.gov.uk/peoplepopulationandcommunity/birthsdeathsandmarriages/deaths/datasets/deathsinvolvingcovid19inthecaresectorenglandandwales; (accessed Oct. 12, 2021)). For 2020-05-15 (the closest available date to 2020-07-14), there were 41135 total deaths for England (= 43168 (England and Wales) - 2033 (Wales)) from which we subtracted 15160 deaths of care home residents ("England, ONS Data") to obtain 25975. For 2020-09-25 (the closest available date to 2020-09-26), there were 50589 total deaths for England from which we subtracted 19880 deaths of care home residents ("England, ONS Data") to obtain 30709. Data for the proportion aged over 65 is for England from 2019 as listed by LG Inform (see https://tinyurl.com/4vhrb2uu; accessed on April 29, 2021). GDP value is obtained by taking the average of values listed in the OECD database for the regions of "South West England", "North East England", "East of England", "North West England", and "South East England". Population value is from the Office of National Statistics report: "Population estimates for the UK, England and Wales, Scotland and Northern Ireland: mid-2020" (see Table 2) (https://www.ons.gov.uk/peoplepopulationandcommunity/populationandmigration/populationestimates/bulletins/annualmidyearpopulationestimates/mid2020; accessed July 30, 2021).

- For Pagani et al. [58] ("Castiglione d'Adda, Italy"), data was taken directly from the paper. Pagani et al. [58] does not cite a specific source for deaths but notes: "From the 1st of January to the 31st of March 2020, 76 deaths (1.65% of the population) have been recorded in CdA, of which 47 were officially attributed to COVID-19." We have not been able to verify these numbers using a publicly available dataset. With regards top LTC deaths, Costantino Pesatori, the mayor of Castiglione D'Adda, is quoted as saying: "In our retirement home alone, we have had at least seven casualties." ("Sono morti tanti anziani, è vero. Solo nella nostra casa di riposo abbiamo avuto almeno sette perdite." See La Stampa: https://www.lastampa.it/topnews/primo-piano/2020/03/16/news/la-strage-silenziosa-di-castiglione-d-adda-43-morti-in-tre-settimane-1.38599650; accessed Oct. 12, 2021). With this in mind, we subtracted 7 and 8 deaths from the total of 47 cited in the paper. Note that it appears that 100% of residents in the nursing home were evntually infected (see https://www.ilcittadino.it/stories/Cronaca/covid-casa-di-riposo-di-castiglione-tutti-gli-anziani-contagiati_61445_96/). For population, Pagani et al. [58] states that "Castiglione d'Adda (CdA) is a town of 4605 inhabitants (according to data from the local registry office at the time of our

study) [...]". Data for the proportion aged over 65 is from https://www.citypopulation.de/en/italy/localities/lombardia/lodi/09801410001__castiglione_dadda/ (accessed July 27, 2021). GDP value is from OECD database for Lombardy region (ITC4).

- For Petersen et al. [61] ("Faroe Islands, Denmark"), information on deaths for the Faroe Islands was obtained from corona.fo/hagtol, the government information website concerning COVID19 in the Faroe Islands, which indicated zero deaths. GDP value is from value listed for Denmark from OECD database. Data for the proportion aged over 65 is from Index mundi (see https://tinyurl.com/bb2pwrx6; accessed on April 29, 2021) which cites the CIA World Factbook as a source. Population value is from Petersen et al. [61] ("In the Faroe Islands, a geographic isolate of 52,154 inhabitants.").

- For Pollán et al. [63] ("Spain"), data for the number of deaths was obtained from Wikipedia (en.wikipedia.org/wiki/COVID-19_pandemic_in_Spain; accessed on April 28, 2021) which sourced the information from the Centro Nacional de Epidemiología (cnecovid.isciii.es/covid19/). Note that, the number of deaths of for 2020-05-11 of 26,920 (14 days after the start of the sampling window), is actually higher than the number of deaths for 2020-05-25 of 26,834 (14 days after the end of the sampling window). This may be due to a reporting issue which is noted by Wikipedia: "Figures for 2020-05-24 to 2020-06-17 include corrections in the validation of past data from several autonomous communities as a result of the transition to a new surveillance methodology implemented from 2020-05-11." We subtracted 34% from the mortality numbers (26834, and 26920) to account for those deaths that occurred in long-term care facilities. This follows the practice done by Pastor-Barriuso et al. [59] who subtracted 9,909 deaths from a the total of 29,137; see discussion within Pastor-Barriuso et al. [59] for details. GDP value is from value listed for Spain from OECD database. Data for the proportion aged over 65 is from World Bank's World Development Indicators (WDI) data (see https://data.worldbank.org/indicator/SP.POP.65UP.TO.ZS (last updated 2021-03-19)). Population value is from the World Bank for 2020 (https://data.worldbank.org/indicator/SP.POP.TOTL?locations=ES; accessed July 30, 2021).

- For Radon et al. [68] ("Munich, Germany"), the number of deaths was obtained from the official database of the city of Munich (see https://www.muenchen.de/rathaus/Stadtinfos/Coronavirus-Fallzahlen.html; accessed June 22, 2021). Gleich et al. [25] conclude that: "One quarter of all COVID-19 deaths in Munich occurred in the context of nosocomial outbreaks in elderly, chronically ill residents of nursing facilities." Based on this finding, we subtract 25% from the lower bound (292) and the upper bound (1040) to obtain the numbers: 219 and 780. Population data is from

https://web.archive.org/web/20201218195500/https://www.muenchen.de/rathaus/Stadtinfos/Statistik/Bev-lkerung.html (accessed June 22, 2021) and data for those aged over 65 from https://www.muenchen.de/rathaus/dam/jcr:459cce24-3894-4d4d-b144-4185b750a310/LHM.Stat_Faltkarte_2019_englisch.pdf (accessed June 22, 2021). Finally, GDP value is from value listed for Bavaria (GE2) from the OECD database.

- For Reyes-Vega et al. [72] ("Lima, Peru"), note that Peru audited death records and their data was updated on May 31, 2021; see Dyer [20] for details. We used official (post-audit) numbers from the Peru Ministry of Health (MINSA) (see https://www.datosabiertos.gob.pe/dataset/fallecidos-por-covid-19-ministerio-de-salud-minsa/resource/4b7636f3-5f0c-4404-8526; accessed June 22, 2021) for mortality numbers. Note that the target population is Lima Metropolitana, an area that includes both area that includes Peruvian provinces of Lima and Callao. As such, we sum deaths recorded for both Lima and Callao provinces. In order to acknowledge that there is substantial uncertainty in the post-audit mortality numbers, we widened the interval by lowering the lower bound by 10% and increasing the upper bound by 10%. Limited information is available about mortality amongst LTC residents [98]. As such, no LTC-adjustments were made to the mortality numbers. GDP value is from value listed for Lima (PE15) from the OECD database. Data for the population aged over 65 from https://www.citypopulation.de/en/peru/admin/15__lima/ (accessed July 27, 2021). Population value obtained from https://www.minsa.gob.pe/reunis/data/poblacion_estimada.asp (accessed July 27, 2021). Note that Reyes-Vega et al. [72] state that: "The study area has an estimated 10.7 million inhabitants."

- For Richard et al. [73] ("Geneva, Switzerland (B)"), note that this study is excluded from the Chen et al.-based analysis set but included in the Serotracker-based analysis set. For Richard et al. [73], mortality data for the canton of Geneva were obtained from an excel file made publicly available by a Swiss government website at: ge.ch/document/covid-19-donnees-completes-debut-pandemie (accessed on April 28, 2021). According to reporting from mid-Jun 2020 by the canton of Geneva (Département de la sécurité, de l'emploi et de la santé (DSES) - Direction générale de la santé - Service du médecin cantonal), about 45% of deaths were among nursing home residents ("résidents des établissements médico-sociaux (EMS)"); see document at https://www.ge.ch/document/19696/annexe/61. We therefore adjusted the numbers by 45%: 122 (=222-0.45*222) and 156 (= 283-0.45*283). Data for the population and the proportion aged over 65 is from https://www.bfs.admin.ch/bfs/en/home/statistics/regional-statistics/

regional-portraits-key-figures/cantons/geneva.html (accessed on April 28, 2021). GDP value is from value listed for Lake Geneva Region (CH01) from the OECD database.

- For Samore et al. [76] ("Four counties in UT, USA"), data for the number of deaths for the counties of Utah county, Salt Lake county, Davis county, and Summit county, was obtained from the county-level COVID-19 dataset curated by the New York Times available at: github.com/nytimes/covid-19-data (accessed on April 28, 2021). The Salt Lake Tribune reported on July 14, 2020 that 42% of COVID-19 deaths in Utah had been attributed to nursing homes ("According to the state health department, 95 of Utah's 226 COVID-19 deaths have been attributed to the facilities."; see https://www.sltrib.com/news/2020/07/14/utah-nursing-homes-keep/; accessed Oct. 12, 2021). This agrees with analysis by the New York Times (see: https://web.archive.org/web/20200627101803/https://www.nytimes.com/interactive/2020/us/coronavirus-nursing-homes.html; accessed Oct. 12, 2021). We therefore adjusted the mortality number by 42% and obtained 40 (=69-69*0.42) and 97 (=168-168*0.42). Data for the proportion aged over 65 is from https://github.com/JieYingWu/COVID-19_US_County-level_Summaries/blob/master/data/README.md (accessed on April 29, 2021). GDP value is from value listed for the state of Utah, USA (US49) in the OECD database. Population value is from US county-level census data for 2019 for the four counties (636235+1160437+355481+42145) (see https://www.census.gov/data/datasets/time-series/demo/popest/2010s-counties-total.html; accessed July 30, 2021).

- For Santos-Hövener et al. [77] ("Kupferzell, Germany"), data for the number of deaths for Kupferzell, Germany was obtained directly from Santos-Hövener et al. [77] which cites the Robert Koch Institute. Despite efforts, no publicly available dataset was found which could confirm these numbers specific these numbers. Santos-Hövener et al. [77] report that 3 deaths occurred in the town for the period before July, 2020, and do not specify if these were nursing home residents. Santos-Hövener et al. [77] do however specify the ages of those who died: "aged 59, 81 and 91 years." Data for the proportion aged over 65 is from: https://ugeo.urbistat.com/AdminStat/en/de/demografia/eta/kupferzell/20172564/4 (accessed on April 29, 2021). GDP value is from value listed for Baden-Württemberg (DE1) from the OECD database. Population value is from Wikipedia (https://als.wikipedia.org/wiki/Kupferzell; accessed July 30, 2021) which cites the Statistisches Landesamt Baden-Württemberg – Bevölkerung nach Nationalität und Geschlecht am 31. Dezember 2019 (CSV-Datei).

- For Selvaraju et al. [78] (“Chennai, Tamil Nadu, India”), mortality data for the number of deaths for Chennai were obtained from the Greater Chennai Corporation (see http://covid19.chennaicorporation.gov.in/; specifically, numbers published for July 15, 2020 and August 14, 2020 were listed at: https://twitter.com/chennaicorp/status/1283280173132128259 and https://twitter.com/chennaicorp/status/1294140456868052993; accessed June 22, 2021). We multiplied the number recorded for 14 days after the end of the sampling window of 2,384 by a factor of 2.5 (based the upper bound of Mukherjee et al. [50]’s estimated underreporting factor for Tamil Nadu) in order to account for potential underreporting. Note that Ariel Karlinsky has assembled excess mortality data for Chennai (see: https://github.com/akarlinsky/world_mortality/tree/main/local_mortality; accessed August 4, 2021). These data, taking into account the weekly numbers and five-year pre-pandemic trend, suggest an interval of [1344, 2454]. However, we prefer not to use this much narrower interval since the excess mortality data have not been adjusted to account for the potentially substantial underreporting of all-cause deaths (see discussion in Anand et al. [4]). There is insufficient information about deaths of LTC residents in India to make any LTC adjustments; see Rajagopalan et al. [69]. Data for the proportion aged over 65 is from: https://statisticstimes.com/demographics/india/tamil-nadu-population.php (accessed June 22, 2021). GDP value is from value listed for Tamil Nadu (IN33) from the OECD database. Population value is from https://www.populationu.com/cities/chennai-population (accessed June 22, 2021).

- For Sharma et al. [80] (“Delhi, India”), infection rate estimates are based on survey data from round 1 of the study (August 1-7). Data for the number of deaths for Delhi was obtained from Wikipedia (en.wikipedia.org/wiki/COVID-19_pandemic_in_Delhi; accessed on April 28, 2021) which sourced the information from the Delhi State Health Bulletin (https://delhifightscorona.in/). We multiplied the number recorded for 14 days after the end of the sampling window of 4,270 by a factor of 6.3 (based on Mukherjee et al. [50]’s estimated underreporting factor) in order to account for potential underreporting. As such, our interval is relatively wide and should reflect the uncertainty in the true number of deaths: [4,188, 26,901]. There is insufficient information about deaths of LTC residents in India to make any LTC adjustments; see Rajagopalan et al. [69]. Data for the proportion aged over 65 is from Statistics Times for 2011 (http://statisticstimes.com/demographics/india/delhi-population.php; accessed on April 29, 2021). GDP value is from value listed for the National Capital Territory of Delhi (IN03) from the OECD database. Note that we use 18.9 million as the estimated population of Delhi, the number cited by both Sharma et al. [80] and Bhattacharyya et al.

[12], while Chen et al. [19] use a much different number: 30,290,936 (see Table S14 in Supplementary Appendix 2 of Chen et al. [19]).

- Snoeck et al. [83] ("Luxembourg") "recruited a representative sample of the Luxembourgish population" between April 16th and May 5th, and obtained a 95% CI of [1.23%, 2.77%]. Two different 95% CIs, obtained with and without adjustment for age, gender and canton are provided in the paper: [1.23%; 2.67%] and [1.34%; 2.77%]. As such, we record [1.23%; 2.77%] for our IR interval. Data for the number of deaths was obtained from the Our World in Data COVID-19 dataset available at: `ourworldindata.org/coronavirus/country/luxembourg` (accessed on April 28, 2021). Hansen and Dalesio [29] report that: "Of the 507 fatalities from Covid-19 in 2020, 241 had lived in housing offering care for the elderly, the Health Ministry reported to Parliament in January." As such we adjusted the numbers by subtracting 47% to obtain 47 (=89-0.47*89) and 58 (=109-0.47*109). GDP value is from value listed for Luxembourg (LU00) from the OECD database. Data for the proportion aged over 65 is from World Bank's World Development Indicators (WDI) data (see `https://data.worldbank.org/indicator/SP.POP.65UP.TO.ZS` (last updated 2021-03-19)). Population value from World Bank for Luxembourg, 2020 (see `https://data.worldbank.org/indicator/SP.POP.TOTL?locations=LU`; accessed July 30, 2021). We note that, while Arora et al. [6] categorizes the sampling method for the Snoeck et al. [83] study as "stratified probability," Snoeck et al. [83] explains that "the sample of participants was enrolled through the use of a non-probabilistic web panel" and caution that "[t]here are pros and cons of using web panels for surveys," specifically that "online surveys may be biased samples because the respondents are self-selected." Levin et al. [38] specifically identify Snoeck et al. [83] as an example of a "active recruitment" study.

- For Sood et al. [84] ("Los Angeles County, CA, USA"), data for the number of deaths for Los Angeles County, CA was obtained from the County of Los Angeles Public Health for 2020-04-24 (913 total deaths, and 384 deaths in institutional settings "as of 8pm 4/26"; see `https://web.archive.org/web/20200426230946/http://publichealth.lacounty.gov/media/coronavirus/locations.htm`; accessed Oct. 12, 2021) and for 2020-04-28 (1111 total deaths, and 505 in institutional settings "As of 8:00pm 4/29"; see `https://web.archive.org/web/20200501065125/http://publichealth.lacounty.gov/media/coronavirus/locations.htm`; accessed Oct. 12, 2021). To be clear, the County of Los Angeles Public Health reports deaths in institutional settings separately. Sood et al. [84] notes that "Residents of Los Angeles County, California, within a 15-mile (24 km) radius of the testing site were eligible for participation." Data for the proportion aged over 65 is from `https://github.com/JieYingWu/COVID-19_US_County-level_Summaries/blob/master/data/README.md` (accessed on April 29, 2021). GDP value is

from value listed for the state of California, USA, (US06) in the OECD database. Population value is from US county-level census data for 2019 (see https://www.census.gov/data/datasets/time-series/demo/popest/2010s-counties-total.html; accessed July 30, 2021).

- For Statistics Jersey [86] ("Jersey, UK (A)"), data for the number of deaths was obtained from the Government of Jersey website (https://www.gov.je/datasets/listopendata?listname=COVID19DeathsClassification; accessed on April 28, 2021) summing both "probable COVID-19" deaths and "laboratory proven" COVID-19 deaths. According to reporting by the BBC on Apr. 27 2020 (see https://t.co/2mj8YJbMsL?amp=1; accessed Oct. 12, 2021), 7 out of 19 deaths occurred in care homes. With this in mind, we adjust our mortality numbers (27, and 28) by 37% to obtain 17 and 18. Data for the proportion aged over 65 is from https://www.indexmundi.com/jersey/demographics_profile.html (accessed on April 29, 2021). GDP value is from value listed for United Kingdom (GBR) in the OECD database. Population value is from Statistics Jersey [86].

- For Statistics Jersey [87] ("Jersey, UK (B)") data for the number of deaths was obtained from the Government of Jersey website (https://www.gov.je/datasets/listopendata?listname=COVID19DeathsClassification; accessed on April 28, 2021) summing both "probable COVID-19" deaths and "laboratory proven" COVID-19 deaths. According to reporting by the BBC on Apr. 27 2020 (see https://t.co/2mj8YJbMsL?amp=1; accessed Oct. 12, 2021), 7 out of 19 deaths occurred in care homes. With this in mind, we adjust our mortality numbers (31, and 31) by 37% to obtain 20 and 20. Data for the proportion aged over 65 is from https://www.indexmundi.com/jersey/demographics_profile.html (accessed on April 29, 2021). GDP value is from value listed for United Kingdom (GBR) in the OECD database. Population value is from Statistics Jersey [87].

- For Streeck et al. [89], the number of deaths for Gangelt, Kreis Heinsberg, Germany, is directly noted in the Streeck et al. [89] article (n=8 deaths). No publicly available dataset was found, however we did find information on the Gangelt municipal bulletin (see www.gangelt.de/news/226-erster-corona-fall-in-nrw; accessed on April 28, 2021) which suggests that 8 is a reasonable number to consider. According to reporting, Gangelt did not suffer substantial (if any) fatalities in LTC facilities during the study period (MedWatch reports that during the study period: "there were no deaths of patients with Covid-19 in the Katharina-Kasper-Heim [nursing home] - nor in a nursing home with 80 residents, which is located in the Breberen district." see: https://medwatch.de/2020/05/08/wie-manche-virologen-vertrauen-verspielen/; accessed on Oct. 18, 2021). Therefore, we did not subtract any deaths to account for nursing home residents. Note that the seroprevalence estimates for this study are based on the results of antibody tests as well

as the results of PCR tests. As such the 14 day offset may not be as appropriate for this study as compared to other studies that consider only antibody tests. Data for the proportion aged over 65 is from Figure S1 of Streeck et al. [89]. GDP value is from value listed for the North Rhine-Westphalia region (DEA) from the OECD database. Population value is from Streeck et al. [89] ("In the German community of Gangelt (12,597 inhabitants, January 1, 2020)").

- For Stringhini et al. [90] ("Geneva, Switzerland (A)"), mortality data for the canton of Geneva were obtained from an excel file made publicly available by a Swiss government website at: `ge.ch/document/covid-19-donnees-completes-debut-pandemie` (accessed on April 28, 2021). According to reporting from mid-June 2020 by the canton of Geneva (Département de la sécurité, de l'emploi et de la santé (DSES) - Direction générale de la santé - Service du médecin cantonal), about 45% of deaths were among nursing home residents ("résidents des établissements médico-sociaux (EMS)"); see document at `https://www.ge.ch/document/19696/annexe/61`. We adjusted the numbers by 45%: 122 (=222-0.45*222) and 155 (= 281-0.45*281). Data for the proportion aged over 65 is from `https://www.bfs.admin.ch/bfs/en/home/statistics/regional-statistics/regional-portraits-key-figures/cantons/geneva.html`. GDP value is from value listed for Lake Geneva Region (CH01) from the OECD database. Population value is from the Swiss Federal Statistical Office (`https://www.bfs.admin.ch/bfs/en/home/statistics/regional-statistics/regional-portraits-key-figures/cantons/geneva.html`; accessed July 30, 2021).

- For Vos et al. [97], mortality data for the Netherlands was obtained from the National Institute for Public Health and the Environment `https://www.rivm.nl/coronavirus-covid-19/grafieken`; accessed Oct. 12, 2021. The numbers used are those listed under "Aantal overledenen naar datum van overlijden" ("Number of deceased by date of death"). Based on available information (see `https://www.verenso.nl/nieuws/archief/2020/update-registratie-verpleeghuizen-12-mei-2020` (accessed Oct. 12, 2021)) there were 1696 nursing home residents who died as of May 12, 2020. This represents about 30% of all reported COVID-19 deaths in the country (1696/5738) up until May 12, 2020. As such we will adjust our numbers by 30% : 2621 (=3744-3744*0.30) and 4186 (=5980-5980*0.30). The GDP value is from data listed for Netherlands (NLD) from the OECD database. Data for the proportion aged over 65 is also from World Bank's World Development Indicators (WDI) data (see `https://data.worldbank.org/indicator/SP.POP.65UP.TO.ZS` (last updated 2021-03-19)). Population value is from the World Bank (`https://data.worldbank.org/indicator/SP.POP.TOTL?locations=NL`; accessed July 30, 2021).

- For Ward et al. [100] ("England, UK (B)"), infection rate estimates are based on survey data from from the first survey (20 June - 13 July). We used mortality numbers obtained from the Office of National Statistics, dataset ("deaths registered weekly in England and Wales"; see publishedweek532020.xlsx, sheet "UK - Covid-19 - Weekly occurrences") retrieved from `https://www.ons.gov.uk/peoplepopulationandcommunity/birthsdeathsandmarriages/deaths/datasets/weeklyprovisionalfiguresondeathsregisteredinenglandandwales` (accessed Oct. 12, 2021). We subtracted those deaths attributed to Wales, and subtracted deaths attributed to nursing home residents as recorded in the "Deaths involving COVID-19 in the care sector, England and Wales" dataset (available at `https://www.ons.gov.uk/peoplepopulationandcommunity/birthsdeathsandmarriages/deaths/datasets/deathsinvolvingcovid19inthecaresectorenglandandwales`; (accessed Oct. 12, 2021)). For 2020-07-03 (the closest available date to 2020-07-04), there were 48718 total deaths for England (= 51207 (England and Wales) - 2489 (Wales)) from which we subtracted 19192 deaths of care home residents ("England, ONS Data") to obtain 29526. For 2020-07-31 (the closest available date to 2020-07-27), there were 49593 total deaths for England from which we subtracted 19555 deaths of care home residents ("England, ONS Data") to obtain 30038. Note that the numbers of cumulative deaths for England, from the UK coronavirus dashboard (`https://coronavirus.data.gov.uk/details/deaths?areaType=nation&areaName=England`; accessed on April 29, 2021) are somewhat different. For example, for 2020-07-04, the UK coronavirus dashboard reports 47,608 deaths for England "with COVID-19 in the death certificate" and 36,233 deaths for England that occurred "within 28 days of a positive test." Data for the proportion aged over 65 is for England from 2019 as listed by LG Inform (see `https://tinyurl.com/4vhrb2uu`; accessed on April 29, 2021). GDP value is obtained by taking the average of values listed in the OECD database for the regions of "South West England", "North East England" "East of England", "North West England" and "South East England". Population value is from the Office of National Statistics report: "Population estimates for the UK, England and Wales, Scotland and Northern Ireland: mid-2020"' (see Table 2 at: `https://www.ons.gov.uk/peoplepopulationandcommunity/populationandmigration/populationestimates/bulletins/annualmidyearpopulationestimates/mid2020`; accessed July 30, 2021).

- For Yoshiyama et al. [102] ("Tokyo, Japan"), for the number of deaths for "2020-06-15" and "2020-06-21", we used official numbers from the Japanese Ministry of Health, Labour, and Welfare (see: `https://www.mhlw.go.jp/content/10906000/000640012.pdf` and `https://www.mhlw.go.jp/content/10906000/000641965.pdf`; accessed August 6, 2021). Estévez-Abe and Ide [21] report: "As of May 8, only 14%

of COVID-19-related deaths in Japan had occurred in LTCFs (Kyodo News Service, 2020).” We adjusted the numbers accordingly: 270 (=314-0.14*314) and 275 (=320-0.14*320). Data for the population and the proportion of the population aged over 65 is from the Tokyo metropolitan government (https://www.metro.tokyo.lg.jp/tosei/hodohappyo/press/2021/01/28/01.html and https://www.metro.tokyo.lg.jp/ENGLISH/ABOUT/HISTORY/history03.htm; accessed August 6, 2021) and GDP value is obtained by taking the average of values listed in the OECD database for the regions of “Northern-Kanto, Koshin” (JPC) and “Southern-Kanto” (JPD).

S1.4 MCMC details and R code

Note that, in order to improve the MCMC mixing, we replace the binomial distribution for CC_k as described in (1), with

$$CC_k \sim Binom(T_k, IR_k), \tag{1}$$

for $k = 1, \ldots, K$. For any sufficiently large P_k, this simplification will make little to no difference. Then, since the distributions of C_k and $D_k|C_k$ are both binomials (see (2) and (3)), we have that unconditionally:

$$D_k \quad \sim Binom(P_k, IFR_k \times IR_k). \tag{2}$$

Note that Z_{1k} is set equal to the centred and scaled logarithm of $65yo_k$, and Z_{2k} is set equal to the centred and scaled logarithm of GDP_k.

The following JAGS-code is used in R for the analysis. See the JAGS user manual [62] for details about writing JAGS code (and also Qi et al. [66] for details about the specification of censored data in JAGS and the alternative modeling strategy necessary for correctly calculating the DIC).

```
library("rjags")
metaIFR <- "model {
# Priors:
icloglog_theta0 ~ dbeta(0.3, 30);
icloglog_beta  ~ dbeta(1, 3);
theta0 <- log(-log(1-icloglog_theta0));
beta  <- log(-log(1-icloglog_beta));
inv.var_sig   <- (1/sigma)^2 ;
inv.var_tau   <- (1/tau)^2 ;
sigma     ~ dnorm(0, 1/10) T(0,);
tau     ~ dnorm(0, 1/10) T(0,);
theta1 ~ dnorm(0, 1/10);
theta2 ~ dnorm(0, 1/10);

# Likelihood:
for(k in 1:K){
cc[k] ~ dbin(ir[k], tests[k]);
    censor.index[k] ~ dinterval(deaths[k], c(deaths_lower[k], deaths_upper[k]))
deaths[k] ~ dbin(ifr[k]*ir[k], pop[k]);
cloglog(ir[k]) <- cloglog_ir[k];
cloglog(ifr[k]) <- cloglog_ifr[k];
cloglog_ir[k] ~ dnorm(beta, inv.var_sig);
cloglog_ifr[k] ~ dnorm(theta0 + theta1*Z1[k] + theta2*Z2[k], inv.var_tau);
  }}"
```

S1.5 Additional Figures

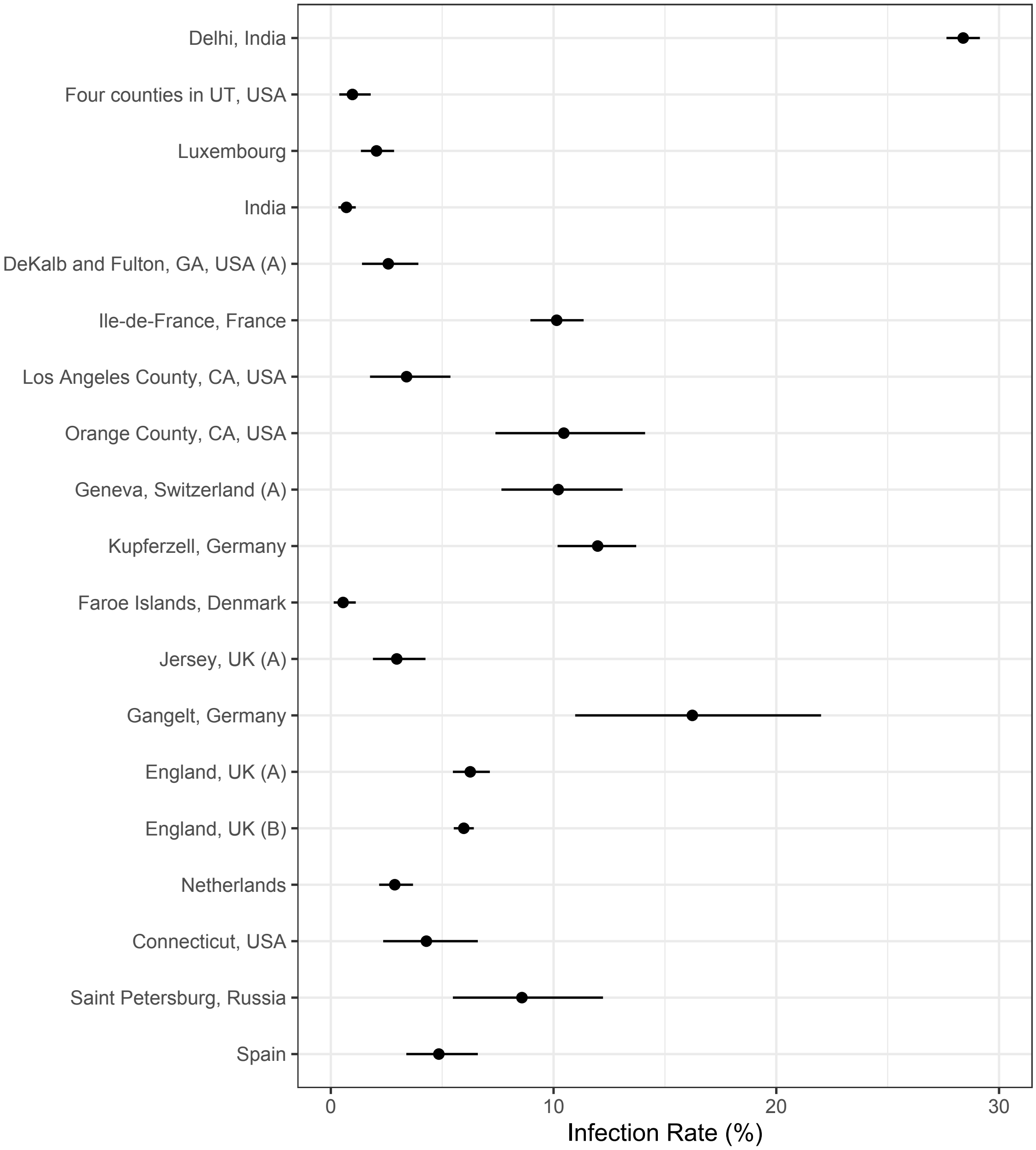

Figure S1: Analysis results from the Chen et al.-based analysis: posterior median estimates for the IR_k variables (for $k = 1, \ldots, 19$) with 95% HPD CrIs. Studies are listed from top to bottom in the same order as in Figure 3.

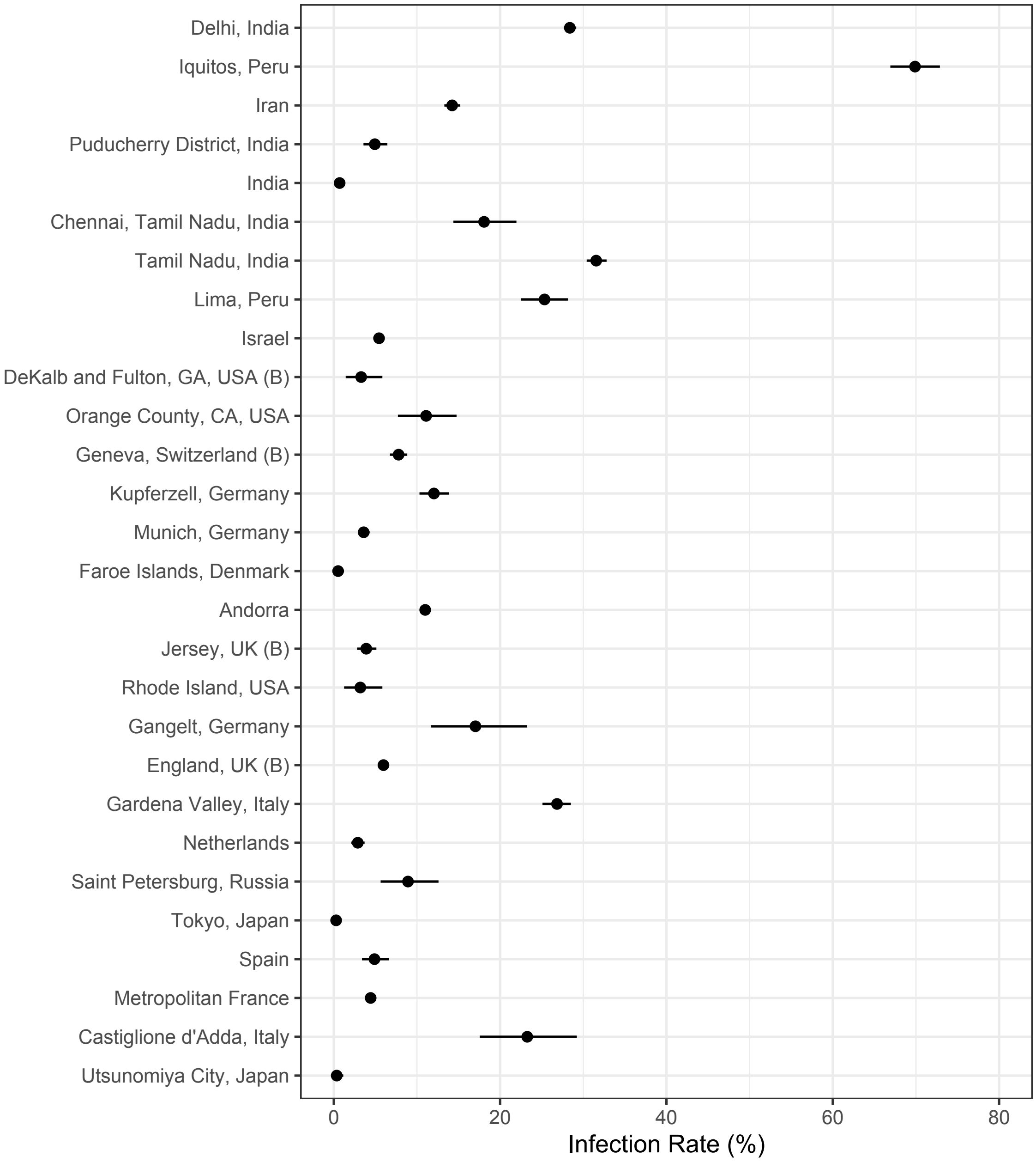

Figure S2: Analysis results from the Serotracker-based analysis: posterior median estimates for the IR_k variables (for $k = 1, \ldots, 28$) with 95% HPD CrIs. Studies are listed from top to bottom in the same order as in Figure 4.

S1.6 Sensitivity analyses

As a sensitivity analysis, we repeated both analyses with an alternative set of priors. For these alternative analyses, we used: $\mathrm{g}^{-1}(\theta_0) \sim Uniform(0,1)$; $\mathrm{g}^{-1}(\beta) \sim Uniform(0,1)$; $\theta_1 \sim \mathcal{N}(0,100)$; $\theta_2 \sim \mathcal{N}(0,100)$; $\sigma \sim$ half-$\mathcal{N}(0,100)$ and $\tau \sim$ half-$\mathcal{N}(0,100)$. The results are plotted in Figures S5 and S6.

We also conducted alternative analyses excluding studies for which the mortality data were not obtained directly from official and reliable sources. See results in Figures S3 and S4. Without the excluded studies, we are unable to provide a reasonable "World" estimate (see the extremely wide credible intervals). However, the "USA" and "EU" estimates are relatively similar.

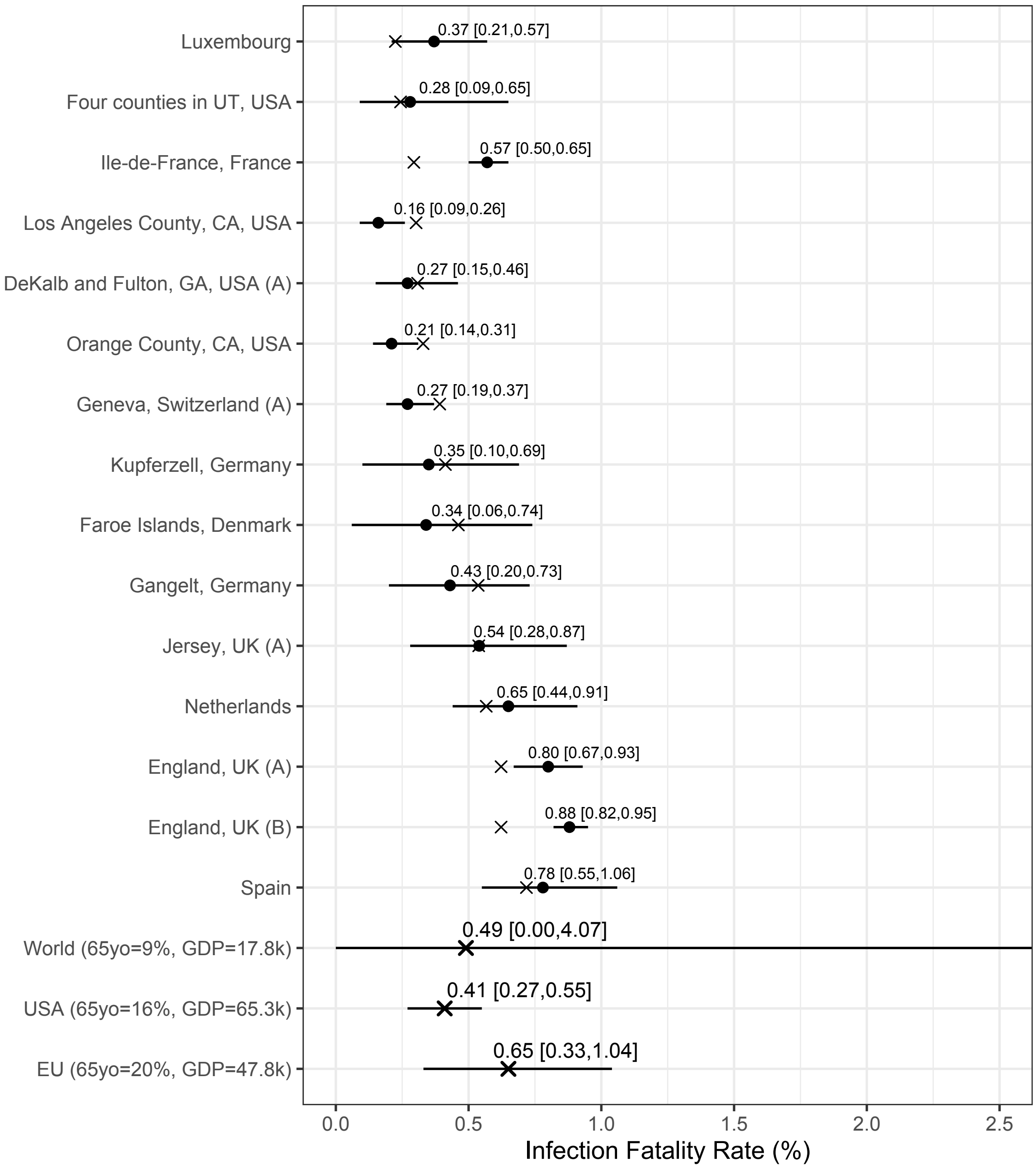

Figure S3: Chen et al.-based analysis results with only those studies for which mortality data was obtained from official sources known to be reliable: posterior median estimates (black circles) for the IFR_k variables (for $k = 1, \ldots, 15$) with 95% HPD CrIs. Studies are listed from top to bottom in order of increasing fitted values (these values are indicated by $\times$). Also plotted, under the labels "World (65 yo=9%, GDP=17.8k)", "USA (65 yo=16%, GDP=65.3k)", "EU (65 yo=20%, GDP=47.8k)", are the posterior median estimate and 95% HPD CrIs for the typical IFR corresponding to values for the proportion of the population aged 65 years and older of 9% and for GDP per capita of $17,811 (the worldwide values), of 16% and of $65,298 (the USA values), and of 20% and of $47,828 (the EU values).

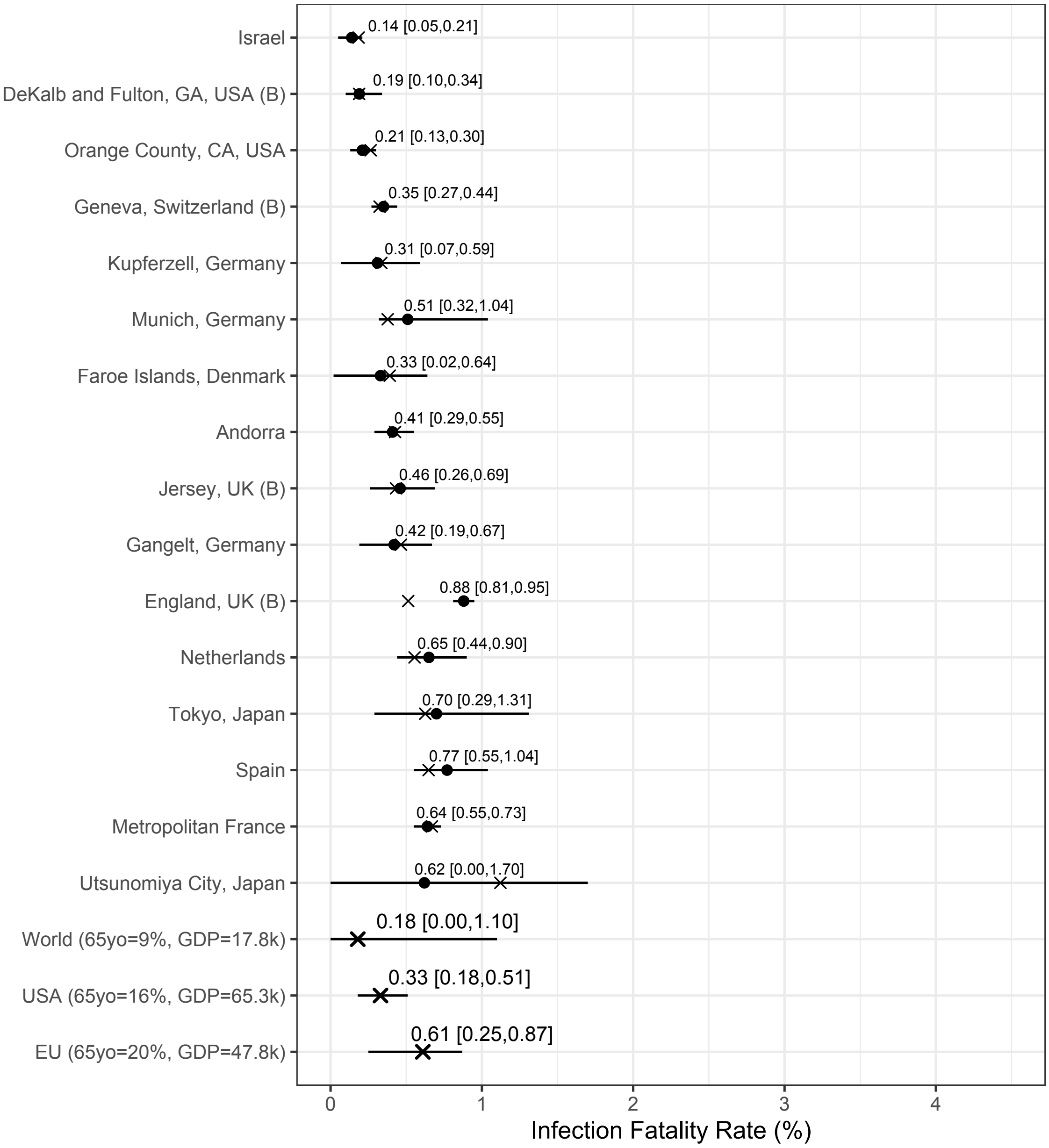

Figure S4: Serotracker-based analysis results with only those studies for which mortality data was obtained from official sources known to be reliable: posterior median estimates (black circles) for the IFR_k variables (for $k = 1, \ldots, 16$) with 95% HPD CrIs. Studies are listed from top to bottom in order of increasing fitted values (these values are indicated by $\times$). Also plotted, under the labels “World (65 yo=9%, GDP=17.8k)”, “USA (65 yo=16%, GDP=65.3k)”, “EU (65 yo=20%, GDP=47.8k)”, are the posterior median estimate and 95% HPD CrIs for the typical IFR corresponding to values for the proportion of the population aged 65 years and older of 9% and for GDP per capita of $17,811 (the worldwide values), of 16% and of $65,298 (the USA values), and of 20% and of $47,828 (the EU values).

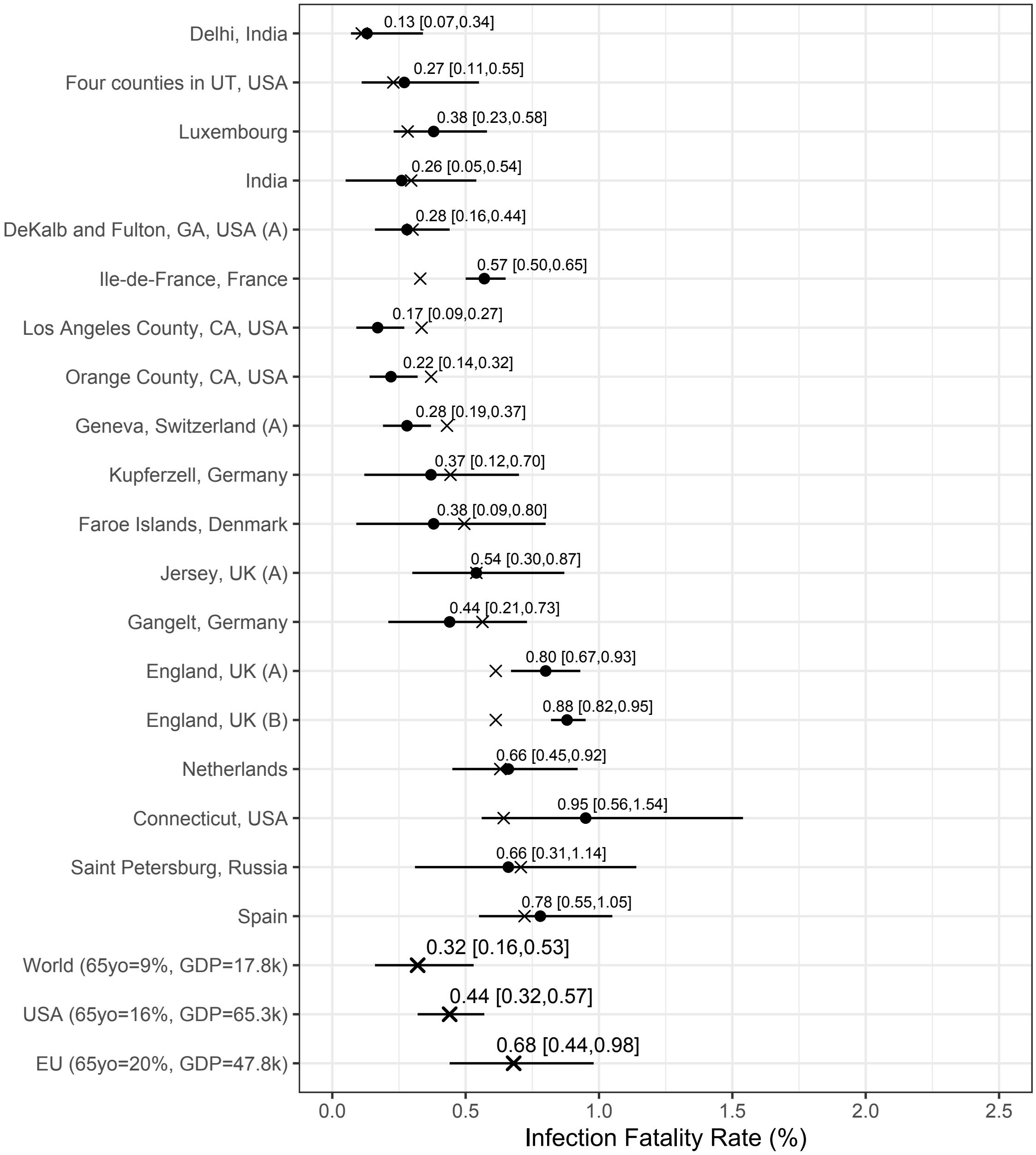

Figure S5: Analysis results from the Chen et al.-based analysis with alternative priors: posterior median estimates (black circles) for the IFR_k variables (for $k = 1, \ldots, 19$) with 95% HPD CrIs. Studies are listed from top to bottom according to increasing fitted values (these values are indicated by $\times$). Also plotted, under the labels "World (65 yo=9%, GDP=17.8K)", "USA (65 yo=16%, GDP=65.3K)", "EU (65 yo=20%, GDP=47.8K)", are the posterior median estimate and 95% HPD CrIs for the typical IFR corresponding to values for the proportion of the population aged 65 years and older of 9% and for GDP per capita of $17,811 (the worldwide values), of 16% and of $65,297 (the USA values), and of 20% and of $47,828 (the EU values).

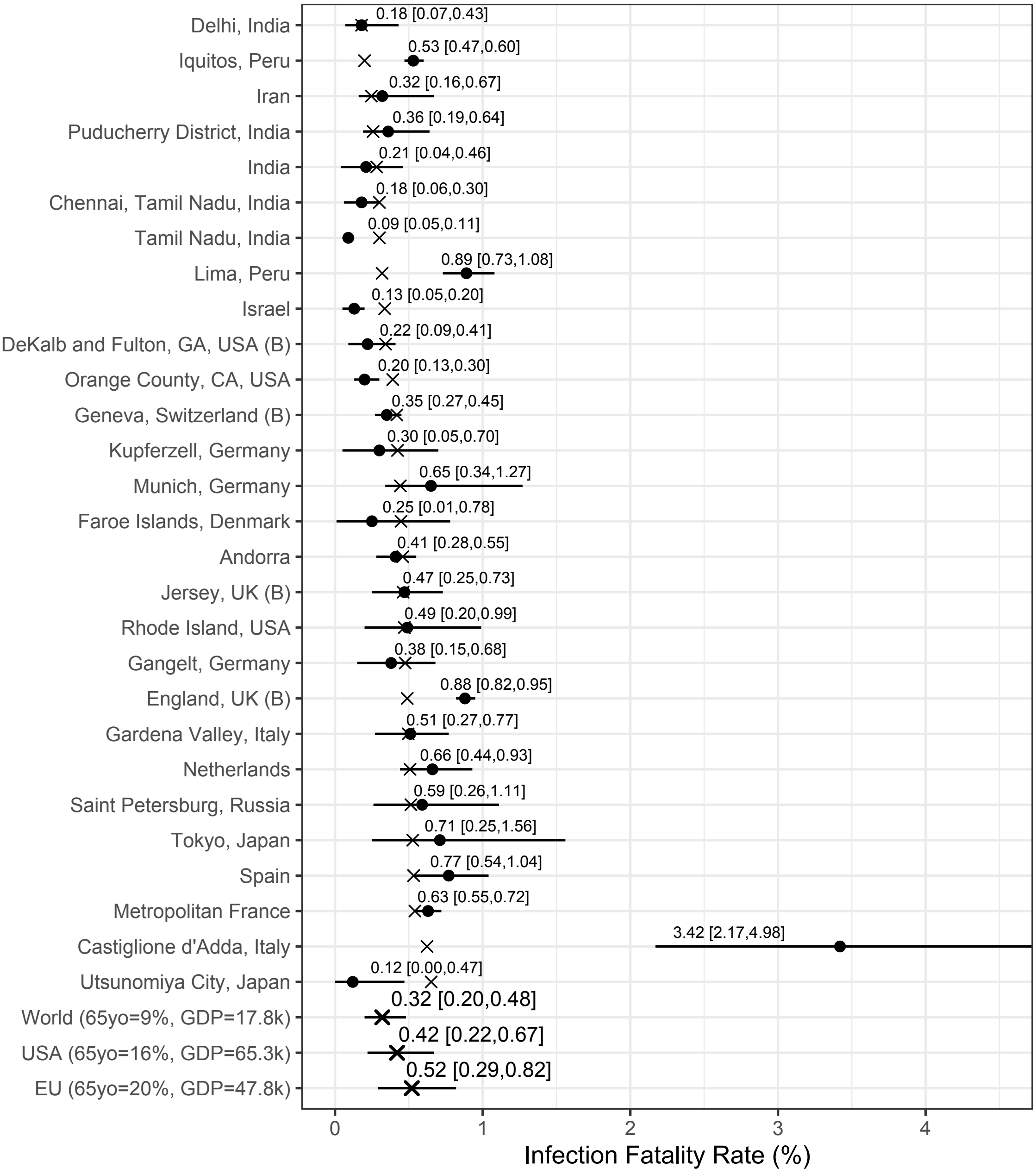

Figure S6: Analysis results from the Serotracker-based analysis with alternative priors: posterior median estimates (black circles) for the IFR_k variables (for $k = 1, \ldots, 28$) with 95% HPD CrIs. Studies are listed from top to bottom according to increasing fitted values (these values are indicated by $\times$). Also plotted, under the labels "World (65 yo=9%, GDP=17.8K)", "USA (65 yo=16%, GDP=65.3K)", "EU (65 yo=20%, GDP=47.8K)", are the posterior median estimate and 95% HPD CrIs for the typical IFR corresponding to values for the proportion of the population aged 65 years and older of 9% and for GDP per capita of $17,811 (the worldwide values), of 16% and of $65,297 (the USA values), and of 20% and of $47,828 (the EU values).

References

[1] Berhanu Alemu et al. Sero-prevalence of anti-SARS-CoV-2 antibodies in Addis Ababa, Ethiopia. *bioRxiv*, 2020. doi:10.1101/2020.10.13.337287.

[2] Álvarez-Antonio et al. Seroprevalence of anti-SARS-CoV-2 antibodies in Iquitos, Loreto, Peru. *medrxiv*, 2021.

[3] Álvarez-Antonio et al. Seroprevalence of anti-sARS-CoV-2 antibodies in Iquitos, Peru in July and August, 2020: a population-based study. *The Lancet Global Health*, 9(7):e925–e931, 2021.

[4] Abhishek Anand et al. Three new estimates of India's all-cause excess mortality during the COVID-19 pandemic. *Center for Global Development - Working Paper*, 2021.

[5] Ayesha Appa et al. Universal PCR and antibody testing demonstrate little to no transmission of SARS-CoV-2 in a rural community. In *Open Forum Infectious Diseases*, 2020.

[6] Rahul K Arora et al. Serotracker: a global SARS-CoV-2 seroprevalence dashboard. *The Lancet Infectious Diseases*, 21(4):e75–e76, 2021.

[7] Insa Backhaus et al. First results of the SERODUS study ("erste ergebnisse der SERODUS-studie"). Institute for Medical Sociology report: `https://www.uniklinik-duesseldorf.de/patienten-besucher/klinikeninstitutezentren/institut-fuer-medizinische-soziologie/das-institut/forschung/serodus`, accessed October 25, 2021, 2021.

[8] Kristina L Bajema et al. Comparison of estimated severe acute respiratory syndrome coronavirus 2 seroprevalence through commercial laboratory residual sera testing and a community survey. *Clinical Infectious Diseases*, 2020.

[9] Anton Barchuk et al. Seroprevalence of SARS-CoV-2 antibodies in Saint Petersburg, Russia: a population-based study. *medRxiv*, 2020.

[10] Adeline Beaumont et al. High seroprevalence of anti-sARS-CoV-2 antibodies after the first wave of the COVID-19 pandemic in a vulnerable population in Perpignan, France. *medRxiv*, 2021.

[11] Eran Bendavid et al. COVID-19 antibody seroprevalence in Santa Clara County, California. *medRxiv*, 2020.

[12] Rupam Bhattacharyya et al. Incorporating false negative tests in epidemiological models for SARS-CoV-2 transmission and reconciling with seroprevalence estimates. *Scientific Reports*, 11(1):1–14, 2021.

[13] Holly M Biggs et al. Estimated community seroprevalence of SARS-CoV-2 antibodies—two Georgia counties, April 28–May 3, 2020. *Morbidity and Mortality Weekly Report*, 69(29): 965, 2020.

[14] Lysandro Borges et al. Seroprevalence of SARS-CoV-2 IgM and IgG antibodies in an asymptomatic population in Sergipe, Brazil. *Revista Panamericana de Salud Pública*, 44, 2020.

[15] Tim A Bruckner et al. Estimated seroprevalence of SARS-CoV-2 antibodies among adults in Orange County, California. *Scientific Reports*, 11(1):1–9, 2021.

[16] Mireia G Carrasco et al. Assessment of the andorran response to SARS-CoV-2 pandemic. *Enfermedades Emergentes*, 20(2):61–70, 2021.

[17] Fabrice Carrat et al. Seroprevalence of SARS-CoV-2 among adults in three regions of france following the lockdown and associated risk factors: a multicohort study. *medRxiv*, 2020.

[18] Philip A Chan et al. Seroprevalence of SARS-CoV-2 antibodies in Rhode Island from a statewide random sample. *American Journal of Public Health*, 111(4):700–703, 2021.

[19] Xinhua Chen et al. Serological evidence of human infection with SARS-CoV-2: a systematic review and meta-analysis. *The Lancet Global Health*, 9(5):E598–E609, 2021.

[20] Owen Dyer. COVID-19: Peru's official death toll triples to become world's highest. *The BMJ*, 373(n1442), 2021.

[21] Margarita Estévez-Abe and Hiroo Ide. COVID-19 and Japan's small death toll in long-term care facilities. *Journal of Aging and Social Policy*, 2021.

[22] Barbara Fraser. COVID-19 strains remote regions of peru. *The Lancet*, 395(10238):1684, 2020.

[23] Anne Gégout-Petit et al. Seroprevalence of SARS-CoV-2, symptom profiles and seroneutralization during the first COVID-19 wave in a suburban area, France. *medRxiv*, 2021.

[24] Shabnam Ghasemyani et al. Components of elderly long-term care system in Iran and selected countries: A comparative study. *Health Scope*, 10(3), 2021.

[25] Sabine Gleich et al. COVID-19 deaths among residents of inpatient nursing homes in munich-causes and places of death. *Bundesgesundheitsblatt, Gesundheitsforschung, Gesundheitsschutz*, 2021.

[26] Govern d'Andorra. Els tests d'anticossos permeten diagnosticar 78 positius de la COVID-19, que podrien haver contagiat unes 360 persones. available at `https://tinyurl.com/dybe27uk` (accessed October 25, 2021), 2020.

[27] Daniel F Gudbjartsson et al. Humoral immune response to SARS-CoV-2 in Iceland. *The New England Journal of Medicine*, 383(18):1724–1734, 2020.

[28] Pedro C Hallal et al. SARS-CoV-2 antibody prevalence in Brazil: results from two successive nationwide serological household surveys. *The Lancet Global Health*, 8(11):e1390–e1398, 2020.

[29] Yannick Hansen and Emery P. Dalesio. Covid deaths continue among elderly in care homes. Luxembourg Times, at: `https://www.luxtimes.lu/en/luxembourg/covid-deaths-continue-among-elderly-in-care-homes-6058bf8cde135b923604daba`, 2021.

[30] Zhenyu He et al. Seroprevalence and humoral immune durability of anti-SARS-CoV-2 antibodies in Wuhan, China: a longitudinal, population-level, cross-sectional study. *The Lancet*, 397(10279):1075–1084, 2021.

[31] Charles Huamaní, Lucio Velasquez, Sonia Montes, Ana Mayanga-Herrera, and Antonio Bernabe-Ortiz. Population-based seroprevalence of SARS-CoV-2 antibodies in a high-altitude setting in Peru. *Available at SSRN 3760458*, 2021.

[32] Sitanshu Sekhar Kar et al. Prevalence and time trend of SARS-CoV-2 infection in Puducherry, India, August–October 2020. *Emerging Infectious Diseases*, 27(2):666, 2021.

[33] Ariel Karlinsky and Dmitry Kobak. Tracking excess mortality across countries during the COVID-19 pandemic with the world mortality dataset. *eLife*, 10:e69336, June 2021. ISSN 2050-084X. doi:10.7554/eLife.69336. URL `https://doi.org/10.7554/eLife.69336`.

[34] Kazem Khalagi et al. Prevalence of COVID-19 in Iran: Results of the first survey of the Iranian COVID-19 serological surveillance program. *Clinical Microbiology and Infection*, 2021.

[35] Jahidur Rahman Khan et al. Healthcare capacity, health expenditure, and civil society as predictors of COVID-19 case fatalities: A global analysis. *Frontiers in Public Health*, 8:347, 2020.

[36] S Khan et al. Results of a population-based survey to estimate the seroprevalence of SARS-CoV-2 specific IgG antibodies in Kashmir, India, seven months after the appearance of the first COVID-19 case. Available at SSRN: `https://ssrn.com/abstract=3820632` or `http://dx.doi.org/10.2139/ssrn.3820632` (accessed October 25, 2021), 2021.

[37] Dmitry Kobak. Excess mortality reveals Covid's true toll in Russia. *Significance*, 18(1):16, 2021.

[38] Andrew T Levin et al. Assessing the age specificity of infection fatality rates for COVID-19: systematic review, meta-analysis, and public policy implications. *European Journal of Epidemiology*, pages 1–16, 2020.

[39] Zhongjie Li et al. Antibody seroprevalence in the epicenter Wuhan, Hubei, and six selected provinces after containment of the first epidemic wave of COVID-19 in China. *The Lancet Regional Health-Western Pacific*, 8:100094, 2021.

[40] Ruijie Ling et al. Seroprevalence and epidemiological characteristics of immunoglobulin M and G antibodies against SARS-CoV-2 in asymptomatic people in Wuhan, China. *China (6/15/2020)*, 2020.

[41] Jiangmei Liu et al. Excess mortality in Wuhan city and other parts of china during the three months of the COVID-19 outbreak: findings from nationwide mortality registries. *The BMJ*, 372, 2021.

[42] Mahajan et al. SARS-CoV-2 infection hospitalization rate and infection fatality rate among the non-congregate population in Connecticut. *The American Journal of Medicine*, 2021.

[43] Mahajan et al. Seroprevalence of SARS-CoV-2-specific IgG antibodies among adults living in Connecticut: Post-infection prevalence (PIP) study. *The American Journal of Medicine*, 134(4):526–534, 2021.

[44] Hussaini Majiya et al. Seroprevalence of COVID-19 in Niger State. *medRxiv*, 2020.

[45] Anup Malani et al. Seroprevalence of SARS-CoV-2 in slums versus non-slums in Mumbai, India. *The Lancet Global Health*, 9(2):e110–e111, 2021.

[46] Valerio Marra and Miguel Quartin. A Bayesian estimate of the COVID-19 infection fatality rate in Brazil based on a random seroprevalence survey. *International Journal of Infectious Diseases*, 2021. doi:https://doi.org/10.1016/j.ijid.2021.08.016.

[47] Colleen C McLaughlin et al. High community SARS-CoV-2 antibody seroprevalence in a ski resort community, Blaine County, Idaho, US. preliminary results. *medRxiv*, 2020.

[48] Roberto Melotti et al. Prevalence and determinants of serum antibodies to SARS-CoV-2 in the general population of the Gardena Valley. *medRxiv*, 2021.

[49] MoHoI Ministry of Health of Israel. National sero-epidemiological coverage for COVID-19. `https://www.gov.il/BlobFolder/reports/de-covid19-28062020-17092020/he/files_publications_corona_DE-covid19.pdf` (accessed October 25, 2021), 2020.

[50] Bhramar Mukherjee et al. Estimating the infection fatality rate from SARS-CoV-2 in India. Available at SSRN 3798552, 2021.

[51] Manoj V Murhekar et al. SARS-CoV-2 antibody seroprevalence in India, August–September, 2020: findings from the second nationwide household serosurvey. *The Lancet Global Health*, 9(3):e257–e266, 2021.

[52] Murhekar et al. Prevalence of SARS-CoV-2 infection in India: Findings from the national serosurvey, May-June 2020. *Indian Journal of Medical Research*, 152(1):48, 2020.

[53] Murhekar et al. SARS-CoV-2 antibody prevalence in India: Findings from the second nationwide household serosurvey, August-September 2020. *The Lancet Global Health*, 9(3): E257–E266, 2020.

[54] Vivek Naranbhai et al. High seroprevalence of anti-SARS-CoV-2 antibodies in Chelsea, Massachusetts. *The Journal of Infectious Diseases*, 222(12):1955–1959, 2020.

[55] Nobutoshi Nawa et al. Seroprevalence of sARS-CoV-2 IgG antibodies in Utsunomiya City, Greater Tokyo, after first pandemic in 2020 (u-corona): a household-and population-based study. *medRxiv*, 2020.

[56] Muhammad Imran Nisar et al. Serial population-based sero-surveys for COVID-19 in low and high transmission. *medRxiv*, 2020.

[57] Office of National Statistics. Coronavirus (COVID-19) infection survey: England. https://www.ons.gov.uk/peoplepopulationandcommunity/healthandsocialcare/conditionsanddiseases/datasets/coronaviruscovid19infectionsurveydata, 2020.

[58] Gabriele Pagani et al. Prevalence of SARS-CoV-2 in an area of unrestricted viral circulation: Mass seroepidemiological screening in Castiglione d'Adda, Italy. *PloS one*, 16(2):e0246513, 2021.

[59] Roberto Pastor-Barriuso et al. SARS-CoV-2 infection fatality risk in a nationwide seroepidemiological study. *medRxiv*, 2020.

[60] Mayte Perez-Olmeda et al. Evolution of antibodies against SARS-CoV-2 over seven months: experience of the nationwide seroprevalence ENE-COVID study in Spain. *medRxiv*, 2021.

[61] Maria Skaalum Petersen et al. Seroprevalence of SARS-CoV-2–specific antibodies, Faroe Islands. *Emerging Infectious Diseases*, 26(11):2760, 2020.

[62] Martyn Plummer. JAGS Version 4.0.0 user manual, 2015.

[63] Marina Pollán et al. Prevalence of SARS-CoV-2 in Spain (ENE-COVID): a nationwide, population-based seroepidemiological study. *The Lancet*, 396(10250):535–544, 2020.

[64] Hossein Poustchi et al. SARS-CoV-2 antibody seroprevalence in the general population and high-risk occupational groups across 18 cities in iran: a population-based cross-sectional study. *The Lancet Infectious Diseases*, 21(4):473–481, 2021.

[65] Soumik Purkayastha et al. Estimating the wave 1 and wave 2 infection fatality rates from SARS-CoV-2 in India. *medRxiv*, 2021.

[66] Xinyue Qi, Shouhao Zhou, and Martyn Plummer. A note on Bayesian modeling specification of censored data in JAGS. *arXiv preprint arXiv:2012.02074*, 2020.

[67] Nouar Qutob et al. Seroprevalence of SARS-CoV-2 in the West Bank region of Palestine: a cross-sectional seroepidemiological study. *BMJ Open*, 11(2):e044552, 2021.

[68] Katja Radon et al. Protocol of a population-based prospective COVID-19 cohort study Munich, Germany (koco19). *medRxiv*, 2020.

[69] Jayeeta Rajagopalan et al. The COVID-19 Long-Term Care situation in India. available at `https://ltccovid.org/wp-content/uploads/2020/05/LTC-COVID-situation-in-India-30th-May-1.pdf`; accessed Oct. 15, 2021, 2020.

[70] Sudarshan Ramaswamy et al. Cross-sectional study on sero-prevalence of SARS-CoV-2 infection in Jabalpur, Madhya Pradesh, India. *Journal of Communicable Diseases (E-ISSN: 2581-351X & P-ISSN: 0019-5138)*, 53(1):82–88, 2021.

[71] Shay Reicher et al. Nationwide seroprevalence of antibodies against SARS-CoV-2 in Israel. *European Journal of Epidemiology*, pages 1–8, 2021.

[72] Mary F Reyes-Vega et al. SARS-CoV-2 prevalence associated to low socioeconomic status and overcrowding in an LMIC megacity: A population-based seroepidemiological survey in Lima, Peru. *EClinicalMedicine*, 34:100801, 2021.

[73] Aude Richard et al. Seroprevalence of anti-SARS-CoV-2 IgG antibodies, risk factors for infection and associated symptoms in Geneva, Switzerland: a population-based study. *medRxiv*, 2020.

[74] Eli S Rosenberg et al. Cumulative incidence and diagnosis of SARS-CoV-2 infection in New York. *Annals of Epidemiology*, 48:23–29, 2020.

[75] Cristina Royo-Cebrecos et al. Mass SARS-CoV-2 serological screening, a population-based study in the Principality of Andorra. *The Lancet Regional Health-Europe*, 5:100119, 2021.

[76] Matthew Samore et al. SARS-CoV-2 seroprevalence and detection fraction in Utah urban populations from a probability-based sample. *medRxiv*, 2020.

[77] Claudia Santos-Hövener et al. Serology-and PCR-based cumulative incidence of SARS-CoV-2 infection in adults in a successfully contained early hotspot (CoMoLo study), Germany, May to June 2020. *Eurosurveillance*, 25(47):2001752, 2020.

[78] Sriram Selvaraju et al. Population-based serosurvey for severe acute respiratory syndrome coronavirus 2 transmission, Chennai, India. *Emerging Infectious Diseases*, 27(2):586, 2021.

[79] Maryam Shakiba, Seyed Saeed Hashemi Nazari, Fardin Mehrabian, Seyed Mahmoud Rezvani, Zahra Ghasempour, and Abtin Heidarzadeh. Seroprevalence of COVID-19 virus infection in Guilan province, Iran. *medRxiv*, 2020.

[80] Nandini Sharma et al. The seroprevalence and trends of SARS-CoV-2 in Delhi, India: A repeated population-based seroepidemiological study. *medRxiv*, 2020.

[81] Mariângela F Silveira et al. Population-based surveys of antibodies against SARS-CoV-2 in Southern Brazil. *Nature Medicine*, 26(8):1196–1199, 2020.

[82] Mariangela Freitas Silveira et al. Time-dependent decay of detectable antibodies against SARS-CoV-2: A comparison of elisa with two batches of a lateral-flow test. *The Brazilian Journal of Infectious Diseases*, 101601, 2021.

[83] Chantal J Snoeck et al. Prevalence of SARS-CoV-2 infection in the Luxembourgish population: the CON-VINCE study. *medRxiv*, 2020.

[84] Neeraj Sood et al. Seroprevalence of SARS-CoV-2–specific antibodies among adults in Los Angeles County, California, on april 10-11, 2020. *JAMA*, 323(23):2425–2427, 2020.

[85] Nicola A Spiers. Rapid response: Comparing REACT with the ONS Infection Survey. *The BMJ*, 2020. URL `https://www.bmj.com/content/371/bmj.m3850/rr`.

[86] Statistics Jersey. SARS-CoV-2: prevalence of antibodies in Jersey (community survey round 2). *https://tinyurl.com/6htjn3af (accessed October 25, 2021)*, 2020.

[87] Statistics Jersey. SARS-CoV-2: prevalence of antibodies in Jersey (community survey round 3). *https://tinyurl.com/hxd8sadb (accessed October 25, 2021)*, 2020.

[88] Paola Stefanelli et al. Prevalence of SARS-CoV-2 IgG antibodies in an area of northeastern Italy with a high incidence of COVID-19 cases: a population-based study. *Clinical Microbiology and Infection*, 27(4):633–e1, 2021.

[89] Hendrik Streeck et al. Infection fatality rate of SARS-CoV-2 infection in a German community with a super-spreading event. *Nature Communications*, 11(1):5829, 2020. doi:10.1038/s41467-020-19509-y.

[90] Silvia Stringhini et al. Seroprevalence of anti-SARS-CoV-2 IgG antibodies in Geneva, Switzerland (serocov-pop): a population-based study. *The Lancet*, 396(10247):313–319, 2020.

[91] Silvia Stringhini et al. Seroprevalence of anti-SARS-CoV-2 antibodies after the second pandemic peak. *The Lancet Infectious Diseases*, 21(5):600–601, 2021.

[92] Beatriz H Tess et al. SARS-CoV-2 seroprevalence in the municipality of São Paulo, Brazil, ten weeks after the first reported case. *medRxiv*, 2020.

[93] The Economist. COVID-19 deaths in Wuhan seem far higher than the official count. available at: `https://www.economist.com/graphic-detail/2021/05/30/covid-19-deaths-in-wuhan-seem-far-higher-than-the-official-count` (accessed October 25, 2021), 2021.

[94] F Truc and Gianpiero Gervino. The effects of physical distancing and lockdown to restrain SARS-CoV-2 outbreak in the Italian municipality of Cogne. *Available at SSRN 3848514*, 2021.

[95] Sharona Tsadok-Rosenbluth et al. Update on COVID-related mortality in care homes in Israel, 12th October 2020. `https://ltccovid.org/2020/10/14/update-on-covid-related-mortality-in-care-homes-in-israel-12th-october-2020/` (accessed October 25, 2021), 2020.

[96] Agne Ulyte et al. Clustering and longitudinal change in SARS-CoV-2 seroprevalence in school children in the canton of Zurich, Switzerland: prospective cohort study of 55 schools. *The BMJ*, 372, 2021.

[97] Eric R A Vos et al. Nationwide seroprevalence of SARS-CoV-2 and identification of risk factors in the general population of the netherlands during the first epidemic wave. *Journal of Epidemiology & Community Health*, 75(6):489–495, 2021. ISSN 0143-005X. doi:10.1136/jech-2020-215678.

[98] Patrick Alexander Wachholz et al. COVID-19: challenges in long-term care facilities for older adults in hispanic american countries. *Geriatrics, Gerontology and Aging*, 14(4): 259–266, 2020.

[99] Xiaoli Wang et al. A population-based seroprevalence survey of severe acute respiratory syndrome coronavirus 2 infection in Beijing, China. *medRxiv*, 2020.

[100] Helen Ward et al. Declining prevalence of antibody positivity to SARS-CoV-2: a community study of 365,000 adults. *medRxiv*, 2020.

[101] Josiane Warszawski et al. In May 2020, 4.5% of the population of metropolitan France had developed antibodies against SARS-CoV-2. https://drees.solidarites-sante.gouv.fr/sites/default/files/2021-01/er1167-en.pdf (accessed October 25, 2021), 2020.

[102] Takashi Yoshiyama et al. Prevalence of SARS-CoV-2–specific antibodies, Japan, June 2020. *Emerging Infectious Diseases*, 27(2):628, 2021.
